# Mesoscale structures in amorphous silks from a spider’s orb-web

**DOI:** 10.1038/s41598-020-74638-0

**Published:** 2020-10-23

**Authors:** Christian Riekel, Manfred Burghammer, Martin Rosenthal

**Affiliations:** The European Synchrotron, ESRF, CS40220, 38043 Grenoble Cedex 9, France

**Keywords:** Biological techniques, Biophysics, Materials science, Nanoscience and technology

## Abstract

Of the 7–8 silk fibers making up an orb-web only the hierarchical structural organization of semicrystalline radial fibers -composed of major ampullate silk- has been studied in detail, given its fascinating mechanical features. While major ampullate silk’s nanofibrillar morphology is well established, knowhow on mesoscale (> 50–100 nm) assembly and its contribution to mechanical performance is limited. Much less is known on the hierarchical structural organization of other, generally less crystalline fibers contributing to an orb-webs’ function. Here we show by scanning X-ray micro&nanodiffraction that two fully amorphous, fine silk fibers from the center of an orb-web have different mesoscale features. One of the fibers has a fibrillar composite structure resembling stiff egg case silk. The other fiber has a skin–core structure based on a nanofibrillar ribbon wound around a disordered core. A fraction of nanofibrils appears to have assembled into mesoscale fibrils. This fiber becomes readily attached to the coat of major ampullate silk fibers. We observe that a detached fiber has ripped out the glycoprotein skin-layer containing polyglycine II nanocrystallites. The anchoring of the fiber in the coat suggests that it could serve for strengthening the tension and cohesion of major ampullate silk fibers.

## Introduction

**Figure 1:**
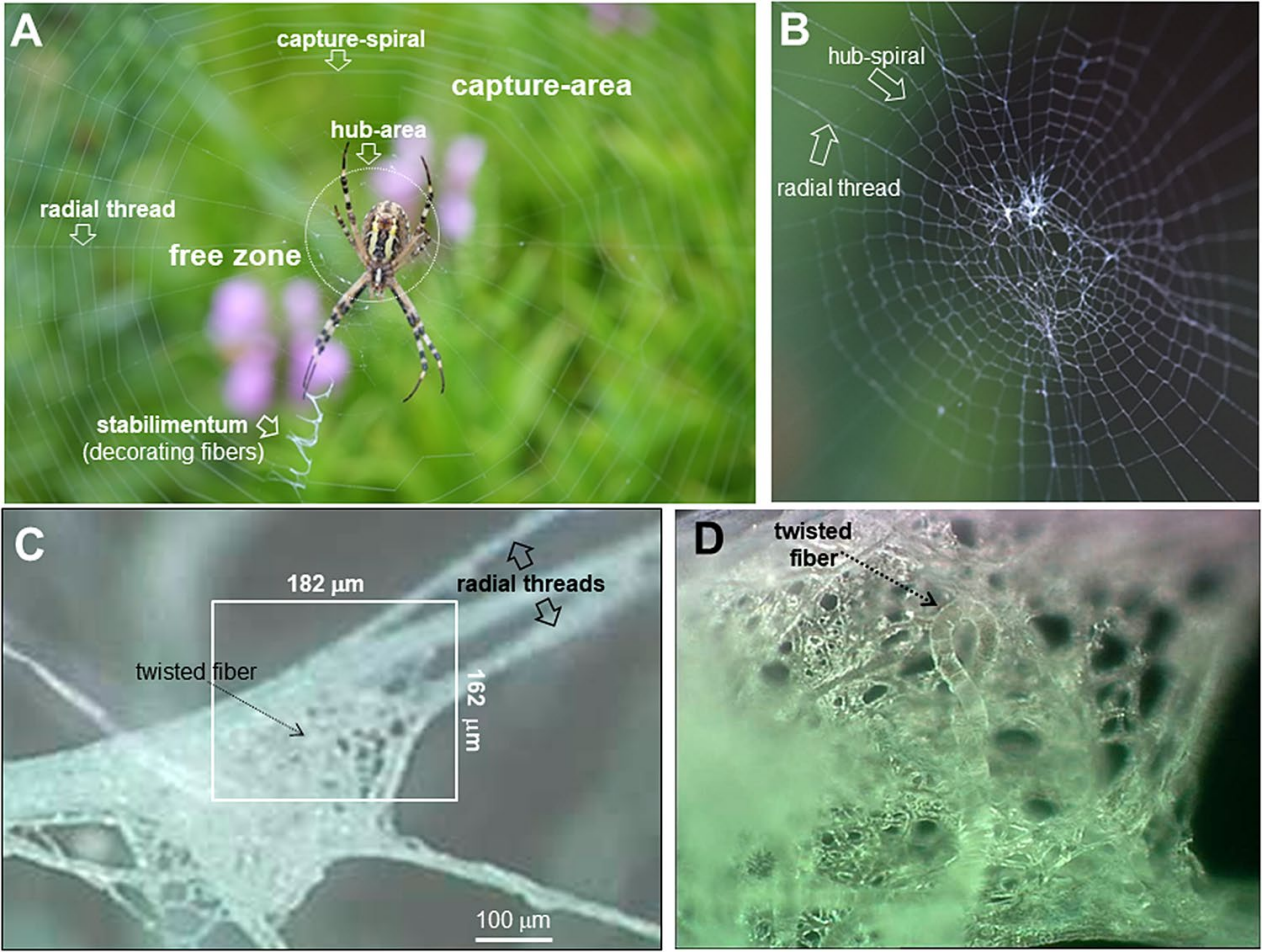
Optical microscopy of orb-web and hub fragment. (**A**) Adult *A. bruennichi* spider located in the hub of its orb-web. For the nomenclature of orb-web parts see:^47^. (**B**) Hub-area from A*. bruennichi’s orb-*web. (**C**) Optical microscopy image of hub-area silk fragment. The rectangular area was probed by scanning microXRD. (**D**) Zoom of rectangular area in (**C**). The twisted fiber indicated by an arrow in (**C**, **D**) is also visible in the SAXS/WAXS-CIs (Fig. 2A,B).

Orb-webs built by *Araneoidea* spiders are composed of seven to eight functional silk fibers (Fig. 1A) ^1, 2^. Of these, the hierarchical structural organization of load-bearing radial or dragline fibers, composed principally of major ampullate gland silk (MaS) proteins, has been studied in most detail by scattering, imaging and spectroscopy techniques. Although its blend of strength, extensibility and toughness shows phylogenetic variability attributed to silk protein evolution^3,4^, the hierarchical structural organisation of MaS fibers derived by scattering techniques is highly conserved^5,6^. Indeed, atomic-scale, wide-angle X-ray and neutron scattering (WAXS/WANS) suggest a two-phase system with 10–15% crystalline, alanine-rich β-sheet nanodomains of a few nm diameters^7–10^, randomly dispersed in a disordered, polypeptidic matrix. Diffuse short-range order (SRO) X-ray scattering peaks^8^ from the amorphous phase do not allow differentiating specific (e.g. helical^11^) hydrogen-bonding motifs. Microscopic models for mechanical performance of dry fibers assume that the nanodomains act as reinforcement-nodes of an amorphous network with hydrogen-bonding interactions^12,13^. Nanoscale, small-angle X-ray scattering (SAXS) and related techniques agree to self-assembly of nanodomains with less-ordered chain segments into lamellar stacks of nanofibrils of 5–7 nm diameters^8,10,14,15^, backed by bottom-up molecular modelling^16^. Scanning nanobeam X-ray diffraction (nanoXRD) with down to ~ 40 nm focal spots suggest a homogeneous nanofibrillar morphology as volume fractions of nanofibrils and nanodomains appear to be correlated^17^. We note, however, variability of nanofibrillar dimensions and volume concentrations when comparing XRD to atomic force microscopy (AFM) and transmission/scanning electron microscopy (TEM/SEM) results, implying that the nature and contribution of nanofibrils to mechanical properties are not fully resolved^18^. A notable exception are ultrathin MaS ribbons of the genus *Loxosceles* whose mechanical properties can be directly related to individual nanofibrils^19,20^. There is, however, a lack of scattering or imaging evidence of higher-order (mesoscale: > 50–100 nm) assembly of nanofibrils in MaS fibers although hierarchical network models based on nanofibrillar domains allow simulating mechanical data such as strain-hardening^21^. Indirect evidence for nanofibrillar bundles of ~ 150 nm diameter was obtained by X-ray particle size analysis for more crystalline (44%) bagworm silk while a smaller diameter of ~ 40 nm was proposed for MaS fibers^15^. The assumption of a homogeneous hierarchical structural organisation assumed in most experimental and modelling approaches is, however, biased by fiber-specific mesoscale heterogeneities. Indeed, a skin–core structure has been observed by TEM for *N. clavipes*^22^ and nanoXRD for *A. bruennichi’s* MaS fibers but is absent for *B. mori* fibers^8,17^. Biochemical analysis of *N. clavipes* fibers suggests a sequence of (from outside) lipidic, glycoprotein and proteneous layers and a core with a heterogeneous distribution of MaSp1 and MaSp2 proteins^23^. Various functions have been attributed to the (mesofibrillar^8,24^) proteneous skin-layer such as providing plasticity and confinement for the core^23^ or counterbalancing the stress generated by the nanofibrils in the core^25^. It is, however, fair to say that the phylogenetic origin of the skin–core structure and its contribution to MaS fibers mechanical properties^4^ are currently not understood.

As compared to MaS fibers, the hierarchical structural organization of less crystalline or amorphous orb-web fibers, such as flagelliform silk (flag) fibers forming the cores of the capture-spiral thread^1,2^, is less well explored. Flag fibers appear also to be structurally more diverse, as *N. clavipes* and *A. bruennichi’s* flag fibers are amorphous showing strong SRO scattering^26,27^ while* A. trifasciata, E. fuliginea* and *C. sexcuspidata* flag fibers contain a fraction of crystalline polyglycine II (PG-II) nanodomains^26,28^ of unknown distribution in the matrix. In the absence of network-reinforcing nodes discussed for MaS fibers^12,13^, molecular or super-molecular reinforcement motifs (e.g. β-springs^29^) have been proposed contributing to the blend of toughness and extensibility of flag fibers^30^. Enhancing these functional properties by mesoscale features could provide an evolutionary advantage for stopping larger preys impacting an orb-web while limiting the metabolic cost of making larger diameter fibers. Indeed, a possible enhancement mechanism could be the incorporation of skin–core structures used for improving mechanical performance of synthetic polymers and biomaterials^31^. Silk fiber composites with mesoscale features are, however, not well documented for *Araneoidea*, as also for other types of spider silks although we note the fibrillar composite structure of *A. aurentatia’s* cylindrical gland fibers used for constructing egg cases^32^.

Probing bulk fibrillar features by TEM^32,33^ or atomic force microscopy (AFM)^34^ requires ultra-thin sections and embedding techniques which are prone to artefacts. In the exceptional case of ultra-thin *L. laeta* ribbons, single nanofibrils could, however, be directly visualized by AFM^19^. While not reaching the resolution of TEM and AFM, scanning nanoXRD can reveal fibrils down to the 100 nm scale and below as shown for the skin-layer of whole *A. bruennichi’s* MaS fibers^8,17^. Indeed, nanoXRD has confirmed the amorphous nature of *A. bruennichi’s* flag fibers while *A. marmoreus* flag fibers show an amorphous core and a mesofibrillar skin-layer containing crystalline PG-II nanodomains^27^. A possible relation of the skin–core structure to the nature of prey caught in the web has been suggested^27^ but more flag fibers have to be studied across *Araneoidea* to establish a trend.

Here we *e*xplore by scanning nanoXRD whether highly amorphous silk fibers with weak chain interactions can contain specific mesoscale features suggesting functional differences. Our aim was detecting such fibers in-situ in the natural environment of orb-web fibers. We focused principally on silks from the hub of *A. bruennichi* spiders orb-web serving as central foraging site (Fig. 1A,B). As compared to the geometrically well-defined mesh of the capture-section composed of fibers with known functions such as load-bearing, stiff radial threads and the flexible, sticky capture-spiral, fibers in the centre of the hub are more irregularly arranged (Fig. 1B)^1,2^ and functionally less well understood or even differentiated. Indeed, the function of decorating fibers from the *stabilimentum* attached to the hub is controversial^35^ and the nature of fibers used for adjusting the final tensioning of radial fibers in the hub^2^ remains to be identified.

We used two approaches for analysing nano&mesoscale fiber features: (i) modelling based on microXRD patterns and (ii) imaging by density projections based on scanning nanoXRD combined with modelling. In view of the levels of structural organization (see above) we will assume that single silk fibers are externally homogeneous with diameters down to approximately the micron-range. “Filament” is used for a detached part of a fiber and “fibril” for a fiber sub-structure with an unspecified diameter. The term “nanofibril” is used for diameters up to about 50 nm and “mesofibril” for diameters up to the micron-scale.

## Results and discussion

### Hub-area density projections

**Figure 2:**
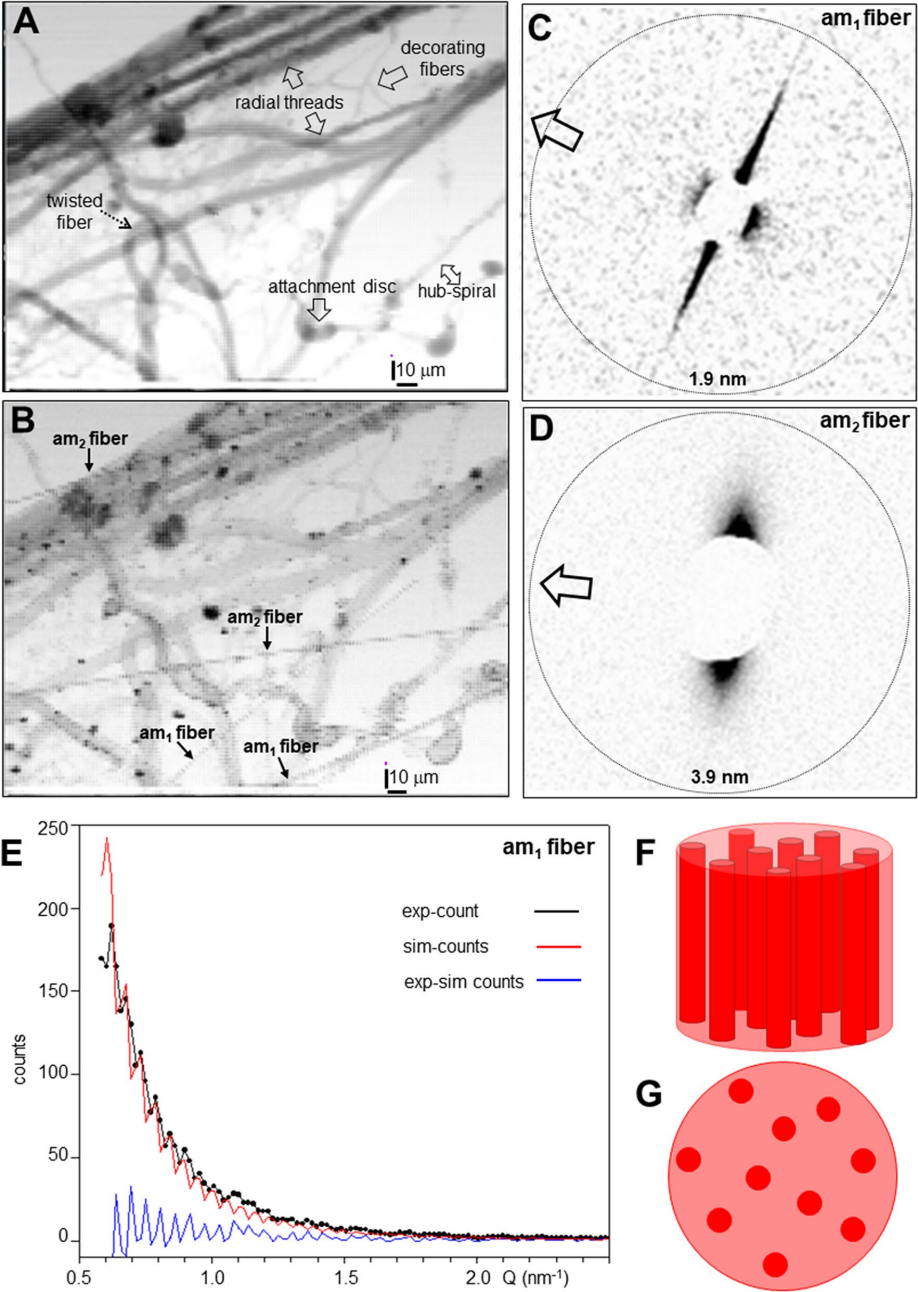
Scanning microXRD and fibrillar composite structure of am_1_ fiber. (**A**) WAXS-CI (angular range of pixels shown in SM Fig. 2A) obtained by scanning microXRD with 2 µm (hxv) step-increments. The twisted fiber is also visible in the optical micrographs (Fig. 1C,D). (**B**) SAXS-CI (angular range of pixels shown in SM Fig. 2B). Clusters of various size on radial threads are due to lipidic deposits^8^. (**C**, **D**) SAXS patterns of am_1_/am_2_ fibers; Open arrow: fiber axis. (**E**) Intensity profile of am_1_ fiber SAXS streak; simulation based on uncorrelated cylinders (SM Eq. 5) with 114.4 nm diameter and smeared by averaging over 5 neighbouring points. Q = 2π/d scale; d: lattice spacing. (**F**, **G**) Schematic model of am_1_ fiber composed of cylinders with about 1/10 of the fibers diameter.

Optical images of the hub fragment reveal a conglomerate of multiple fibers (Fig. 1C,D). We generated composite images (CIs) of the hub fragment by scanning microXRD, corresponding to density projections based on X-ray scattering contrast (see Methods). We identify in the WAXS CI (Fig. 2A) several orb-web features^1^ such as two bunches of four radial threads and the hub-spiral (contrast provided in both cases by Bragg peaks and SRO^8^) as well as the mesh of *decorating silk* fibers (contrast provided principally by SRO^17^). We observe the same features in the SAXS CI (Fig. 2B). The contrast is provided in this case by fibrillar correlations and shape-transforms^8^. Further microstructural data supporting these identifications are presented in the Supplementary Material. We observe, however, only in the SAXS-CI several fine fibers (Fig. 2B). The absence or weakness of Bragg peaks and SRO scattering suggests their highly amorphous nature. The scattering contrast for the amorphous fibers is provided by equatorial SAXS streaks, oriented normal to the fiber axis. We identify two different types of amorphous fibers distinguished by narrow (am_1_) and more diffuse streaks (am_2_) (Fig. 2C,D). In the following we will analyse the hierarchical structural organization of am_1_ fibers based on a microXRD pattern derived from the SAXS-CI. The mechanically particularly stable support provided by a MaS bridge-thread section for an am_2_-type fiber enabled scanning nanoXRD with subµm step increments. This allowed analysing the am_2_ fibers hierarchical structural organization based on real space SAXS&WAXS-CIs and reciprocal space nanoXRD patterns.

### Fibrillar composite structure of am_1_ fiber

The diameter of the projected am_1_ fiber was derived from the spatial distribution of equatorial streaks in the SAXS-CI as 2.0 ± 0.4 µm (SM Fig. 2H,I). The streaks can be related to the transform of fibrillar shapes which can be approximated by cylinders with diameter d_c_ (see Methods). Indeed, the modulated intensity decay of the streak in Fig. 2D was modeled by cylinders of d_c_ ~ 115 nm diameter where the matrix of (assumed) lower density provides the scattering contrast (Fig. 2E; SM Eq. 4). The azimuthal width of the streak translates into an orientation distribution of *f*_*c*_ = 0.998(1) corresponding to quasi-parallel mesofibrils (SM Fig. 3C; SM Eq. 1). The homogeneous distribution of streaks across the line-scans of the fiber (SM Fig. 2I) suggest the model of parallel, cylindrical mesofibrils distributed randomly in the matrix with an undefined volume density (Fig. 2F,G). The presence of meridional diffuse scattering, which cannot be resolved from the beamstop (Fig. 2C), suggests that the model of infinite, homogeneous cylinders is an idealization. The model resembles the structure of egg case silk fibers from *A. aurantia* cylindrical glands^32^, showing stiffness and extensibility comparable to MaS fibers^30,36,37^. The mesoscale features of am_1_ fibers suggest therefore also enhanced stiffness. The lack of an important SRO fraction suggests that molecular contributions to extensibility (e.g. helical motifs) can be excluded. However, while am_1_ fibers have a hierarchical structural organization resembling egg case silk it is not established that they are also produced by cylindrical glands.

**Figure 3:**
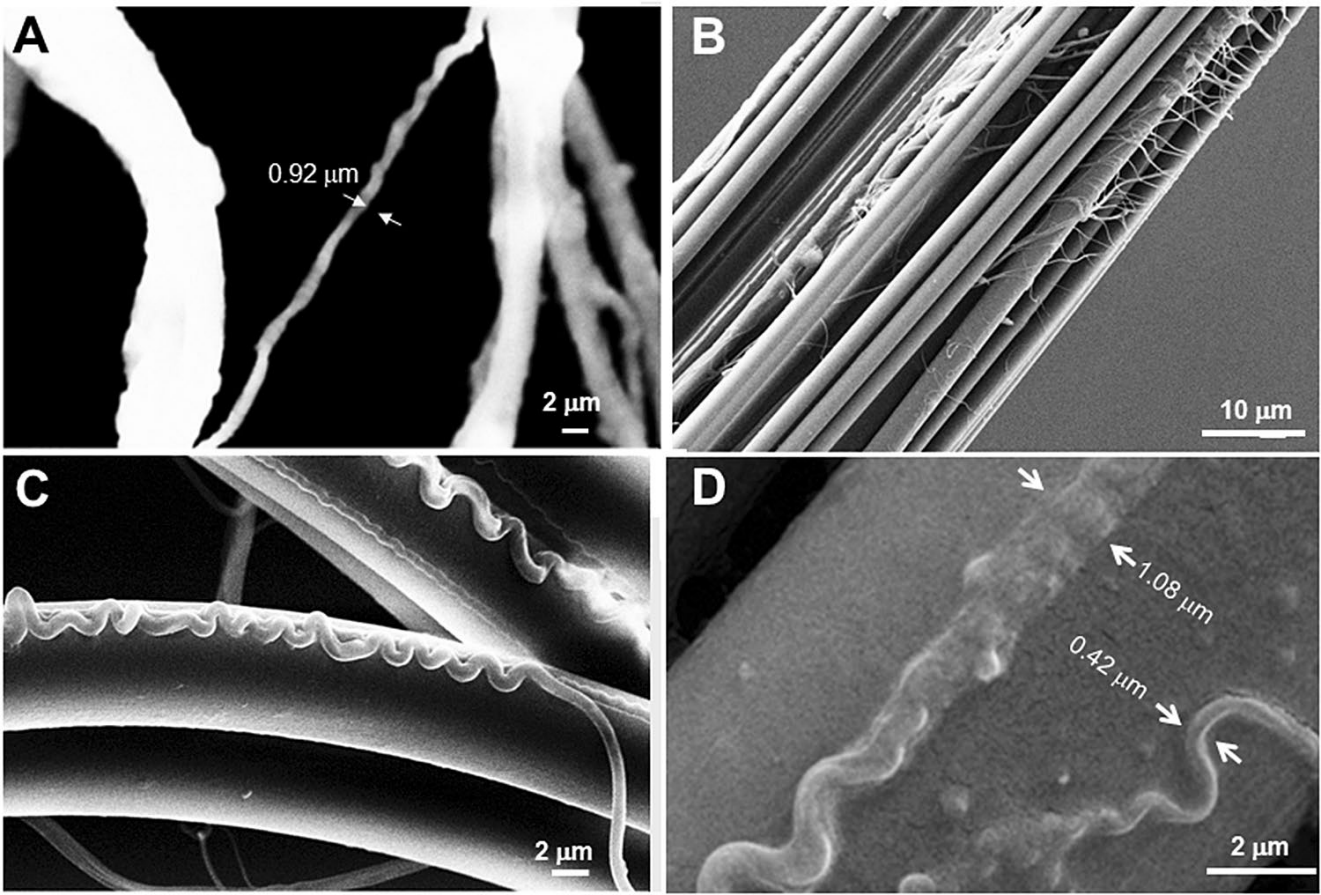
SEM images of MaS and accompanying fibers. (**A**) SEM image of crimped fiber detaching from MaS thread. (**B**) SEM images of bridge-thread fibers with accompanying thin, filamentary fibers. (**C**) Crimped fiber bridging MaS radial thread. (**D**) Zoom into crimped fiber revealing ribbon-like morphology with filamentary substructures, partially fused with the MaS surface. The fibers were obtained from the web fragments studied by micro-/nanoXRD but do not correspond to the features probed (adapted in modified form from^8^; with permission by the authors).

### Skin–core structure of am_2_ fiber

**Figure 4:**
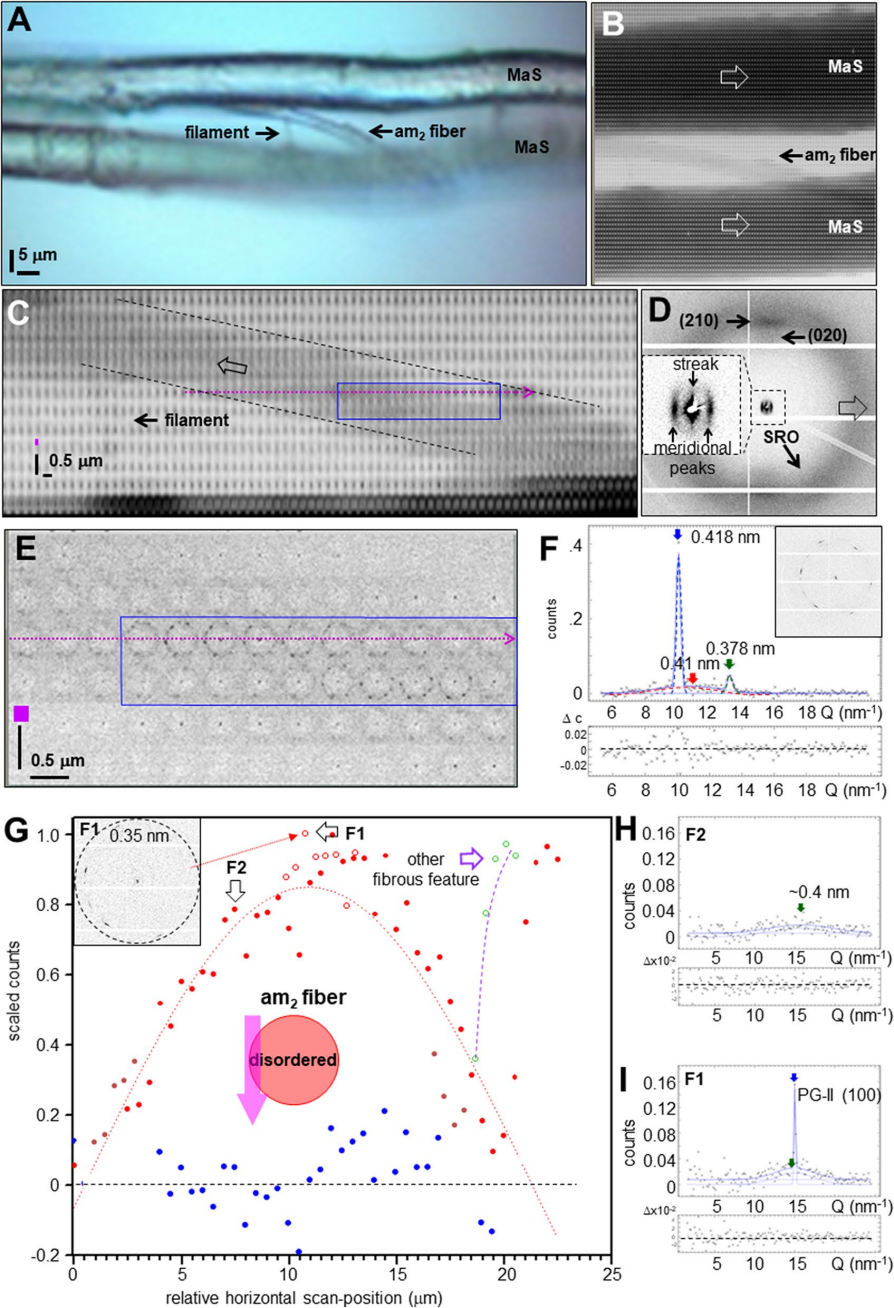
Composite images and structural analysis of am_2_ fiber. (**A**) Optical microscopy of bridge thread and am_2_ fiber. (**B**) NanoXRD WAXS-CI: 0.5 µm (hxv) steps-increments; pixels based on WAXS-range in (**D**), 16 × binned to reduce noise level. Upper intensity level reduced to reveal am_2_ fiber scattering. (**C**) Zoom into WAXS-CI revealing am_2_ fiber. Contours of crystalline domain (blue rectangle) defined by Bragg peaks. (D) Single pixels with Bragg peaks indexed for the poly(L-alanine) lattice^8,48^; Inset: SAXS range with equatorial streak and meridional peak. (**E**) Zoom into crystalline domain. (**F**) Intensity profile from 11 averaged patterns from crystalline domain (inset) fitted by 3 Gaussians for PG-II Bragg peaks^42^ and SRO scattering and a 0-order polynomial for random background (Q = 2π/d scale; d: lattice spacing). (**G**) Integrated diffuse intensity (2 < Q(nm^−1^) < 13.2) of am_2_ fiber along violet, dashed arrow in (**D**,**E**), PG-II (100) peak masked. Red filled circles: am_2_ scattering. Bragg peaks were observed at the position of the red open circles. The green open circles correspond to other nanofibrillar features (see text). Red dashed curve: sine function fitted to the am_2_ data points, green circles were excluded from the fit. An idealized cylindrical cross-section model for the am_2_ fiber and the relative dimensions of the nanobeam is shown as in-set. (**H**) Pattern from position F2 in (**G**) fitted by one Gaussian and a 0-order polynomial). (**I**) Pattern from position F1 in (**G**) fitted by two Gaussians and a 0-order polynomial.

The association of am_2_-type with MaS fibers suggests a functional relation. Indeed, the SAXS-CI obtained by scanning microXRD reveals a crimped am_2_ fiber emanating from the upper bunch of radial threads (Fig. 2B). The separation of a crimped fiber from a MaS thread is also visible in a SEM image (Fig. 3A). A spectrum of crimped and elongated, flexible fibers with ribbon-like features is covering bridge-thread fibers (Fig. 3B,C). Partial fusion of crimped fibers with the MaS skin reveals about 300 nm diameter substructures -limited by the SEM resolution- implying surface interactions (Fig. 3D). The crimped morphology is particularly well visible when the overall am_2_ fiber direction is along the MaS fiber axis (Fig. 3C). The fiber becomes extended when bridging the two fibers of the MaS thread. We generated SAXS&WAXS-CIs based on scanning nanoXRD of an am_2_ fiber attached to a bridge-thread section with a gap between the two fibers (Fig. 4A). The straight, bridging part of the am_2_ fiber has remained connected to one of the MaS fibers by a thin filament (Fig. 4A). This geometry provides stability for scanning nanoXRD and allows observing weak scattering from the am_2_ fiber without scattering from the bridge-thread. Indeed, the WAXS-CI reveals the projection of the am_2_ fiber and the filament by faint diffuse scattering (Fig. 4B,C). The fiber is tilted by 7° ± 1° from the horizontal scan axis, corresponding also to the fiber axis direction of the MaS fibers, revealed by the contrast provided by β-sheet peaks and SRO scattering^8^ (Fig. 4B,D). The am_2_ fiber density projection is, however, not completely homogeneous as we observe within its contours a several µm long domain defined by two Bragg peaks of 0.418 ± 0.001 nm and 0.378 ± 0.004 nm lattice spacings (Fig. 4C,E,F). The nature of this domain will be discussed below. The diameter of the am_2_ fiber was determined from the intensity distribution of a horizontal scan-line (Fig. 4C,E) as for the am_1_ fiber (SM Fig. 2I), assuming that the diffuse scattering at each scan-point (Fig. 4G) is proportional to the amount of protein probed by the nanobeam. Although we masked the strongest (0.418 nm) Bragg peak visible in several patterns (Fig. 4I) we find a slight asymmetry of the profile suggesting enhanced diffuse scattering from an 2nd, unknown component (Fig. 4G). We simulated the profile by a sine function with a width of 10 µm fwb (full-width-at-base) (Fig. 4G) corresponding to a fiber diameter of 1.2 ± 0.2 µm (10 µmxsin7° ± 1°). A sine function is appropriate for the continuous shape-change of a cylindrical cross-section; a hollow cylinder is excluded as the diffuse scattering would increase when probing from the outer skin and decrease towards the center of the cylinder (SM Fig. 4A,B).

**Figure 5:**
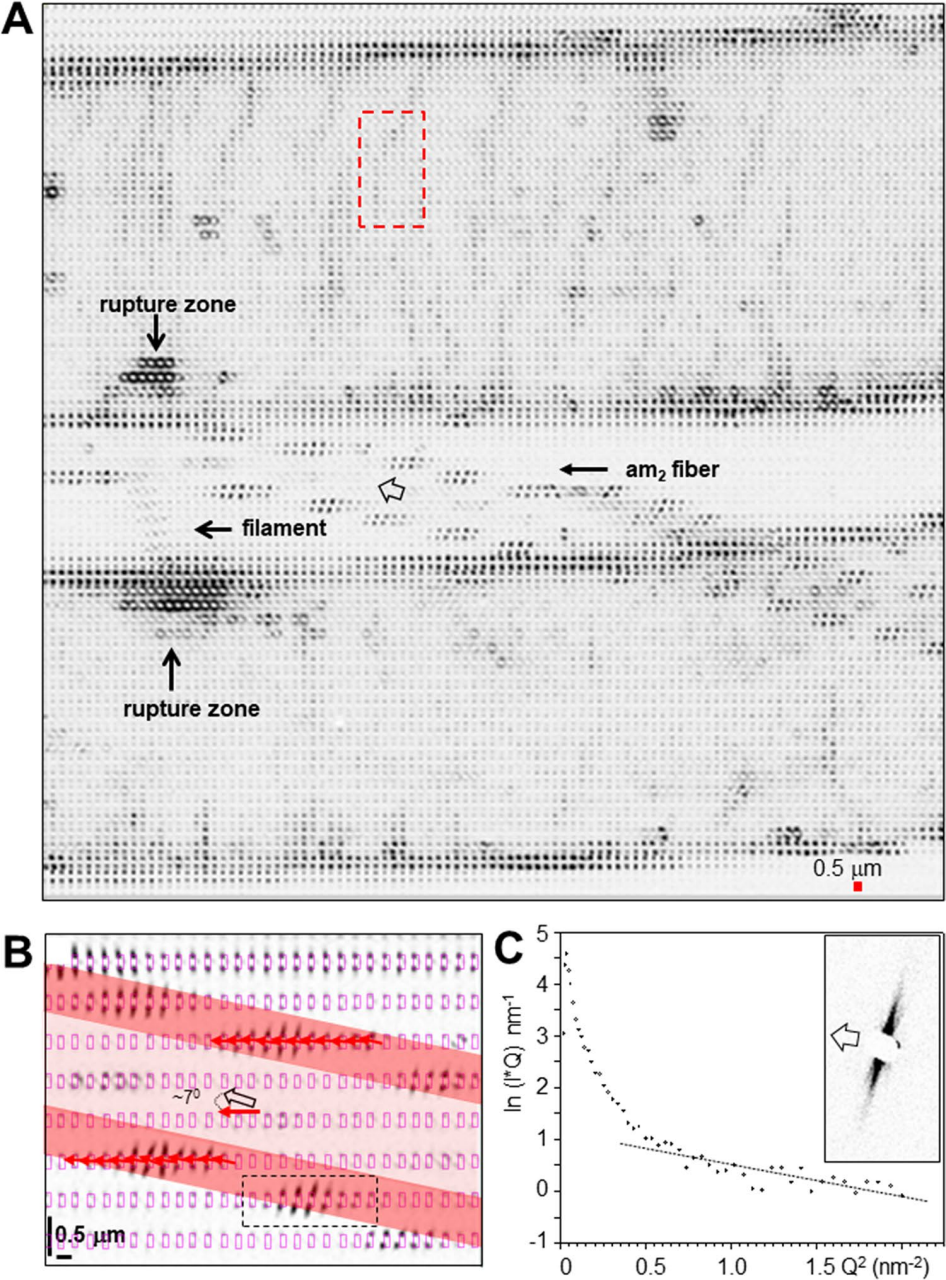
Skin–core structure of am_2_ fiber. (**A**) SAXS-CI based on pixels covering the equatorial streak in Fig. 4D. The contours of the am_2_ fiber in the gap of the MaS thread can be traced by groups of equatorial streaks. (**B**) Zoom of am_2_ fiber with skin-layer defined by groups of horizontal streaks. Aspect ratio ~ 3:1 for better visualization of the fiber contours. The X-ray probing points are shown schematically, scaled to the size of the focal spot. Each 0.5 µm horizontal step corresponds to an incremental vertical displacement of the focal spot across the fiber by 61 ± 9 nm (= 500 nmxsin7° ± 1°). Red arrow: averaged local fibrillar axes direction; black arrow: am_2_ fiber axis. (**C**) Guinier intensity plot for the streak shown in inset. A linear regression line with slope m = −0.61 (0.7) has been fitted to Q^2^ > 0.3 nm^−2^^14^.

**Figure 6:**
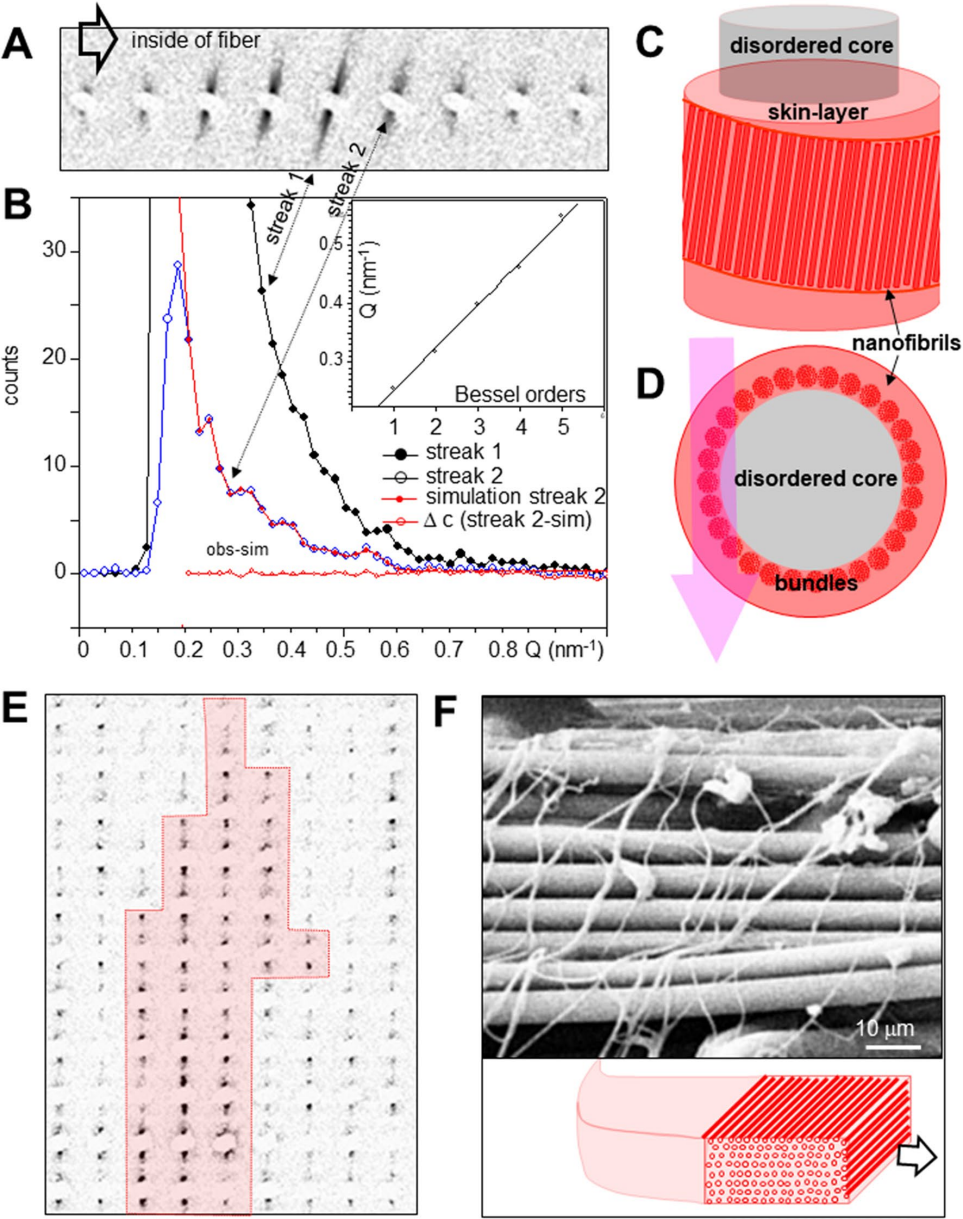
Nanofibrillar am_2_ fiber skin and related filamentary features. (**A**) Evolution of streaks across skin-layer of am_2_ fiber (Fig. 5B). (**B**) Variation of intensity profiles for two streaks from skin-layer. The profile of streak 2 (blue line, open blue circles) has been fitted by 5 Gaussian (red line, solid red circles. The inset shows a correlation plot of peak positions determined by Gaussian fits to the profile versus Q to determine the cylinder radius (text). A regression line with slope m = 0.073 (2) has been fitted. (**C**) Schematic model of am_2_ fiber composed of ribbons of nanofibrils tilted by about 8° against the macroscopic am_2_ fiber axis, wound around a disordered core. Only one layer of nanofibrils in a ribbon (not to scale) is shown. (**D**) Projection of model showing assembly into mesoscale nanofibrillar bundles at the inside of the skin-layer. (**E**) Zoom of red, dashed area in Fig. 5A. Band-like feature indicated by red domain defined by enhanced intensity streaks. (**F**) SEM image of filamentary features covering bridge-thread fibers with schematic fibrillar model for one of the filaments (from same web silk but not from area probed by nanoXRD).

The SAXS-CI is based on the contrast provided by the equatorial streaks (Fig. 4D). The contours of the bridge-thread are revealed by an enhanced contrast of the skin-layer mesofibrils^8^ (Fig. 5A). The banded structure on the bridge-thread will be discussed below. The am_2_ fiber is revealed by horizontal groups of streaks defining a skin–core structure (Fig. 5A,B). The azimuthal width of the strongest streak in a group (e.g. streak 1 in Fig. 6A) corresponds to a spread in fibrillar orientations of about 8° fwhm or about 15° fwhm for an averaged streak of a whole scan-line (SM Fig. 3A,B). Averaging across a larger fiber volume is at the origin of the diffuse streak observed by microXRD (Fig. 2D). Similar groups of streaks define three further fibrous features which are partially overlapping in the am_2_ fibers density projection (SM Fig. 6B). We identify one of these features in the optical micrograph as the filament connecting the am_2_ fiber to the lower MaS fibers surface (Fig. 4A). The other fibrous features in the projection image are optically out of focus. The absence of an equatorial Bragg peak implies the absence of inter-fibrillar correlations. The angular deviation of the fibrillar axis from the direction of the am_2_ fiber axis by ~ 8° is attributed to a wound ribbon of fibrils. The intensity variation in a group of streaks (Figs. 6A, SM Fig. 6B,C) is due to the geometry of the skin-layer probed. Indeed, for a cylindrical cross-section, the nanobeam will probe more fibrils through the skin-layer at the edge than at the center of the fiber (SM Fig. 4A,B). The projection of the fiber diameter derived from the spatial extension of the two groups of streaks in a single horizontal line is 1.2 µm ± 0.2 µm as also derived from the diffuse scattering data (Fig. 4G). The thickness of the skin-layer determined from the extension of the group of streaks is ~ 200 nm. The filament shows also streaks expected for fibrillar morphology (SM Fig. 7A). Albeit the low counting statistics we start seeing a modulation of the intensity profile as for the am_2_ fiber profile suggesting mesofibrils (SM Fig. 7B). We assume therefore that the filament and probably the other fibrous features visible in the SAXS-CI (SM Fig. 6B) have become detached during rupture of the am_2_ fiber from the MaS skin-coat. A cylinder diameter of d_c_ = 4.4 ± 0.3 nm diameter was determined by Guinier’s approach (Supplementary Materials) for the strongest streak showing a continuous intensity decay (Fig. 5C). This diameter corresponds to the scale of nanofibrillar diameters obtained by different techniques. Indeed, diameters of 6–7 nm were obtained Guinier’s approach for nanofibrils in the cores *Nephila* and *Argiope* MaS fibers^8,14^. A diameter of 4.7 nm was derived from the equatorial SAXS correlation peak for bagworm silk nanofibrils^15^ while single nanofibrils with 20 nm × 7 nm cross-section were imaged by AFM for *recluse* spider silk ribbons^19^. The diffuse scattering profile (Fig. 4G) suggests that am_2_ fiber cores contain disordered protein. For the projection of ribbon-like skin-layer one would expect observing towards the center of the fiber fibrillar scattering at different crossing-angles. Indeed, we observe patterns with multiple weak streaks which we attribute to fibrils at discrete angles (SM Fig. 7C,D). The weakness of these streaks does, however, not provide information on the homogeneity of the skin-layer which might have become locally degraded during the rupturing process. Preliminary evidence suggests at least partial assembly of nano- into mesofibrils in the skin-layer. Indeed, a modulation of the equatorial streak profile is observed for the weaker streaks at the inside of the skin-layer (Figs. 6B, SM Fig. 8A). We determined the peak positions by fitting Gaussians to the profile (SM Fig. 8B) and derived a cylinder diameter of d_c_ = 86 ± 3 nm from an extrapolation of the order position versus the scattering vector Q (Fig. 6B) ^8,38^. A slightly larger value of d_c_ ~ 100 nm is obtained by fitting a 1st order Bessel function to the profile (Figs. 6B, SM Fig. 8B; SM Eq. 5). These values are in the range of cylindrical diameters for the mesofibrillar skin-layer of MaS fibers^8^. Mesofibrils could, however, only be observed at the inside of the am_2_ fibers skin-layer as the modulation of streaks from the center and the outside of the skin-layer is not well pronounced. We prefer therefore the idealized model shown in Fig. 6C,D. It is possible that aggregation of nanofibrils is related to mechanical compression at the inner skin-layer. We note that mesofibrillar dimensions and braid-like morphology of am_2_-fibers resemble MaS fibers proteneous skin-layer.

### Banded pattern on bridge-thread

The banded pattern on the surface of the bridge-thread SAXS-CI (Fig. 5A) is generated by streaks with enhanced intensity along the direction of the bands, overlapping with the equatorial streaks from the core nanofibrils^8^. We exclude scattering from “ordered regions” of a few µm size derived from Raman studies for *Nephila* and *B. mori* fibers^39^ as no evidence for bulk crystalline domains of this size was obtained by scanning nanoXRD of *A. bruennichi’s* bridge-threads^8^. We rather attribute the banded pattern to scattering from the mesh of filaments wound around the bridge-thread fibers (Figs. 3B). The density of these filaments appears to fluctuate locally along the threads or even disappear. We have selected a single band by its enhanced scattering (Fig. 6E). Selected streaks from the band show the modulated intensity decay of mesofibrils suggesting equatorial-type scattering (SM Fig. 5A,B). We therefore propose as model layers of mesofibrils which are aligned normal to the axis of the filaments (Fig. 6F). Given the close association of these band-like features and the am_2_-fibers with the MaS-surface (see also below) we put forward the hypothesis of a common glandular origin. Indeed, the proximal region of the MaS gland has been proposed to be at the origin of the MaS fibers core while protein secreted by the distal region was proposed to be at the origin of *Nephila*’s proteneous skin-layer^22^; also assumed for *A. bruennichi*’s MaS fibers^8^. The different fibrous morphologies could therefore be due to different levels of assembly.

### Interaction of am_2_ fiber with MaS fibers coat

We discuss here the nature of the domain observed within the contours of the am_2_ fiber projection, which is defined by Bragg peaks (Fig. 4C,E,F). The peaks show an azimuthal spread which can be separated into several Gaussians suggesting a cluster of nanocrystallites (SM Fig. 9A–D). Slight changes in the texture of the peaks suggest clusters differing slightly in orientation along the am_2_ fiber. The same peaks corresponding to 60–100 nm nanocrystallites were observed for MaS bridge-threads^8^ while we derive 30–40 nm nanocrystallites for the am_2_ fiber domain (Supplementary Material). These nanocrystallites, observed initially only together with MaS diffraction patterns (called S,S* peaks), were explained by the non-periodic lattice (NPL) model based on peptide chain side-group ordering of the β-sheet nanocrystalline fraction^40,41^. A recent model attributes these peaks, however, to the PG-II lattice as they are also observed for the MaS glycoprotein skin-layer^23^ lacking poly(L-alanine) peaks^17^. Peak positions and intensities of the Bragg peaks in the am_2_ domain agree also to PG-II (100)/(101) reflections^42^. We assume therefore that a fragment of the glycoprotein shell containing PG-II nanocrystallites has been ruptured together with the am_2_-fiber section from the MaS coat. Indeed, glycoprotein is revealed by its SRO scattering (Fig. 4H) contributing to the asymmetry of the diffuse scattering profile (Fig. 4G). The SAXS-CI reveals two rupture zones on the MaS thread and the filament connecting the am_2_ fiber to one of the rupture zones (Fig. 5A, SM Fig. 10A–E). Streaks from the MaS proteneous skin-layer with about 15° azimuthal fwhm (similar to the skin of pristine MaS fibers^8^) are observed next to the lower rupture zone (SM Fig. 10D) while random SAXS due is observed within the rupture zone (SM Fig. 10E). We attribute the random SAXS to randomly orientated mesofibrils in the proteneous skin-layer below the ruptured glycoprotein layer. Indeed, the radial profile of the streaks shows the modulated intensity decay of the streaks of the MaS mesofibrillar skin-layer^8^. The scattering data support therefore the biochemical analysis of the MaS coat^23^.

## Conclusions

Our results suggest that even highly amorphous spider silk fibers can contain specific mesoscale features suggesting functional differences. Indeed, we have identified two types of amorphous, fine silk fibers by their mesoscale organizations. The fibrillar composite structure of am_1_ fibers derived from modeling reciprocal space features in microXRD patterns appears providing enhanced stiffness as for egg case silk fibers although we do not have evidence that both types of fibers have the same glandular origin. The ribbon of weakly interacting nanofibrils wound around a core of disordered protein of am_2_ fibers observed in real space by scanning nanoXRD resembles the skin–core structure of MaS fibers^8^ suggesting a blend of toughness and flexibility. The results suggest that am_2_ fibers can be anchored in the glycoprotein skin-layer of MaS fibers enabling functions related to fine-tuning the tension of the radial threads or increasing the lateral cohesion of bridge-thread fibers. It is probable that am_2_-type fibers and other fibrous surface features have the same glandular origin as MaS fibers protein skin-layer suggesting that care is required when interpreting surface-sensitive spectroscopy and imaging techniques.

The evolution of nanoXRD towards sub-100 nm focal spots and enhanced reciprocal space resolution enabled by the upgraded ESRF source^17,43^ should allow refining the am_1_ fiber model by imaging mesofibrils in real space and resolving a possible meridional correlation peak. Probing of fiber sections with smaller focal spots should allow refining the proposed partial assembly of nanofibrils in the am_2_ fibers skin-layer into mesofibrils. It will also be necessary developing protocols for transferring delicate orb-web structures on sample supports while maintaining their architecture in order to reduce background scattering and provide a mechanically stable support for scanning nanoXRD. This would allow generating coarse density projections of larger areas followed by high-resolution scanning of specific features. A general conclusion emerging from this work is that practically any orb-web silk fiber has become accessible to scanning nanoXRD.

## Materials and methods

### Silk fibers

The fibers were obtained from the orb-webs of two adult *Argiope bruennichi* (*Araneidae*) spiders living in their natural habitat (Fig. 1B) and transferred onto Si_3_N_4_ membranes for X-ray scattering analysis^8^. We collected a fragment from the central hub including part of the *stabilimentum*^1^ and a piece from a bridge-thread supporting the orb-web with an attached crimped fiber of unknown glandular origin, also found in the hub-area. The samples were imaged by optical microscopy and SEM. (Supplementary Material).

### X-ray scattering

Scanning microXRD was performed in transmission geometry on the hub-silk sample using a monochromatic SR beam of *λ* = 0.0940 nm wavelength, focused to an about 1.5(h) µm × 1.5(v) µm full-width-half-maximum (fwhm) spot. Scanning nanoXRD was performed in the same geometry on the bridge-thread sample using a monochromatic SR beam of *λ*  = 0.0835 nm, focused to an about 190(h) nm × 170(v) nm (fwhm) spot^8^. A selected area of each sample was mesh-scanned through the focus. Radiation damage by propagating radicals was limited by a step-width of ≥ 500 nm (hxv)^8^. XRD patterns covering lattice spacings from the SAXS to WAXS range were recorded at each mesh-point by an ultrasensitive Si pixel detector.

### Composite X-ray diffraction images

The sequence of patterns from a mesh-scan was transformed into a so-called “composite image” (Abbr.: CI) corresponding to a density projection with a lateral resolution determined by the step-scan increments. “WAXS-CIs” were based on pixels covering the reciprocal space from Q ~ 2 nm^−1^ (d ~ 3.1 nm) to Q ~ 25 nm^−1^ (d ~ 0.25 nm) where: Q = 2π/d scale; d: lattice spacing. “SAXS-CIs” were based on pixels covering reciprocal space from the edge of the beamstop (Supplementary Materials) to ~ 3.3 nm^−1^ (d ~ 1.9 nm). The contrast in SAXS&WAXS-CIs is provided by Bragg peaks, SRO, molecular shapes or nanoscale aggregates^8,17^. Specific features can be localized in CIs based on pixels limited to the corresponding part of reciprocal space such as Bragg peaks or SAXS streaks.

### SAXS/WAXS analysis

Real space information was obtained from patterns by comparison with known patterns or by modelling based on lattices and shapes. Information on extracting atomic-scale information such as lattice metrics, crystallinity, particle size and orientation distribution from Bragg peaks in the WAXS range is provided in the Supplementary Materials. Here we will indicate briefly the approach used for analyzing SAXS features; details are provided in Supplementary Materials. Indeed, SAXS patterns of MaS fibers show generally up to two meridional Bragg peaks due to density modulations along the nanofibrils^8,14^ and an equatorial streak attributed to the nanofibrillar shape transform^10,44^. For amorphous fibers with fibrillar morphology only the equatorial streak is observed. The modulated intensity decay observed for mesofibrils was approximated by uncorrelated cylinders of diameter d_c_ and modelled by a Bessel function (SM Eq. 4, 5). The micro- and nanobeam instrumental resolution provides access to d_c_ ~ 100 nm while the modulation period becomes too extended for d_c_ ≤ 10 nm, i.e. in the range nanofibrils. An approximate d_c_ value was in such a case determined from the slope of the streaks intensity decay by Guinier’s approach^8,10,14^. The equatorial interface scattering streak^45,46^ at the edge of fibers in SAXS-CIs is weak or not observable. We exclude scattering from the interface of cavities which have an irregular distribution in the bulk of MaS fibers^22^ while equatorial SAXS streaks show a regular distribution down to at least the 50 nm scale^17^.

## Supplementary information

Supplementary information
