# Supplementary material for: Mesoscale structures in amorphous silks from a spider’s orb-web
Source: Sci Rep. 2020 Oct 23;10:18205. doi: 10.1038/s41598-020-74638-0 (PMC7584646; doi:10.1038/s41598-020-74638-0)
Supplement: Supplementary file 1 — Supplementary information [file 41598_2020_74638_MOESM1_ESM.pdf]

**Supplementary Material**

# Mesoscale structures in amorphous silks from a spider's orb-web

Christian Riekel[1], Manfred Burghammer[1], and Martin Rosenthal[1]

[1] The European Synchrotron, ESRF, CS40220, F-38043 Grenoble Cedex 9, France

## Materials and Methods

**Silk fibers** were collected from the orb-webs of two adult *Argiope bruennichi (Araneidae)* spiders living in their natural habitat and transferred onto X-ray transparent, low scattering background $Si_3N_4$ membranes without adding water, conservation or fixation agents[1]. A hub-area silk fragment with attached *decorating silk* fibers from the *stabilimentum* was transferred onto a membrane without maintaining its architecture. We also transferred a section of a MaS bridging-thread with an attached fine fiber of unknown glandular origin onto a membrane. The samples were stored at about 4°C prior to data collection.

**Imaging by scanning electron microscopy (SEM) and optical microscopy.** SEM images were recorded at 10 KV using a Zeiss Leo system. A 5 nm Au-layer was deposited by sputtering to avoid charging. Optical microscopy was performed using an inverted Olympus microscope with bright field and epi-illumination.

**Synchrotron radiation (SR) scattering** experiments were performed at the ID13 beamline of the "The European Synchrotron (ESRF)" using a monochromatic beam with $\Delta\lambda/\lambda\sim2*10^{-4}$ band width obtained from the pink beam of an undulator source by a liquid nitrogen cooled double Si crystal. Experiments were performed in transmission geometry with the SR beam oriented approximately normal to the $Si_3N_4$ membrane surface[1,2]. Samples were kept during the experiments at room temperature (about 23 °C), in air and without control of the humidity level.

X-ray microdiffraction (microXRD) was performed with a $\lambda\sim0.094$ nm SR-beam focused to an about 1.5 µm (hxv) (fwhm) spot at the sample position by a composite of parabolic Be refractive lenses[3]. The flux in the focal spot was $\Phi\sim6.4x10^{11}$ photons $s^{-1}$ corresponding to a flux density of $\Phi_d\sim2.9x10^5$ photons $s^{-1}$ $nm^{-2}$. The cut-off by the beamstop corresponded to a lattice spacing of ~14 nm ($Q\sim0.45$ $nm^{-1}$ with $Q=2\pi/d=4\pi\sin\Theta\lambda^{-1}$). The sample-to-detector distance was calibrated by an $Al_2O_3$ powder standard (NBS: SRM 674a) to 203.6 mm.

X-ray nanodiffraction (nanoXRD) was performed with a λ=0.0835 nm SR-beam focused to an about 190(h) nm x 170(v) nm (fwhm) spot at the sample position by Si refractive lenses in crossed geometry. The flux in the focal spot was $\Phi \sim 5x10^9$ photons $s^{-1}$ corresponding to a flux density of $\Phi_d \sim 1.5x10^5$ photons $s^{-1}$ $nm^{-2}$. The cut-off by the beamstop corresponded to a lattice spacing of ~25 nm. The sample-to-detector distance was calibrated by an $Al_2O_3$ powder standard to 253.3 mm.

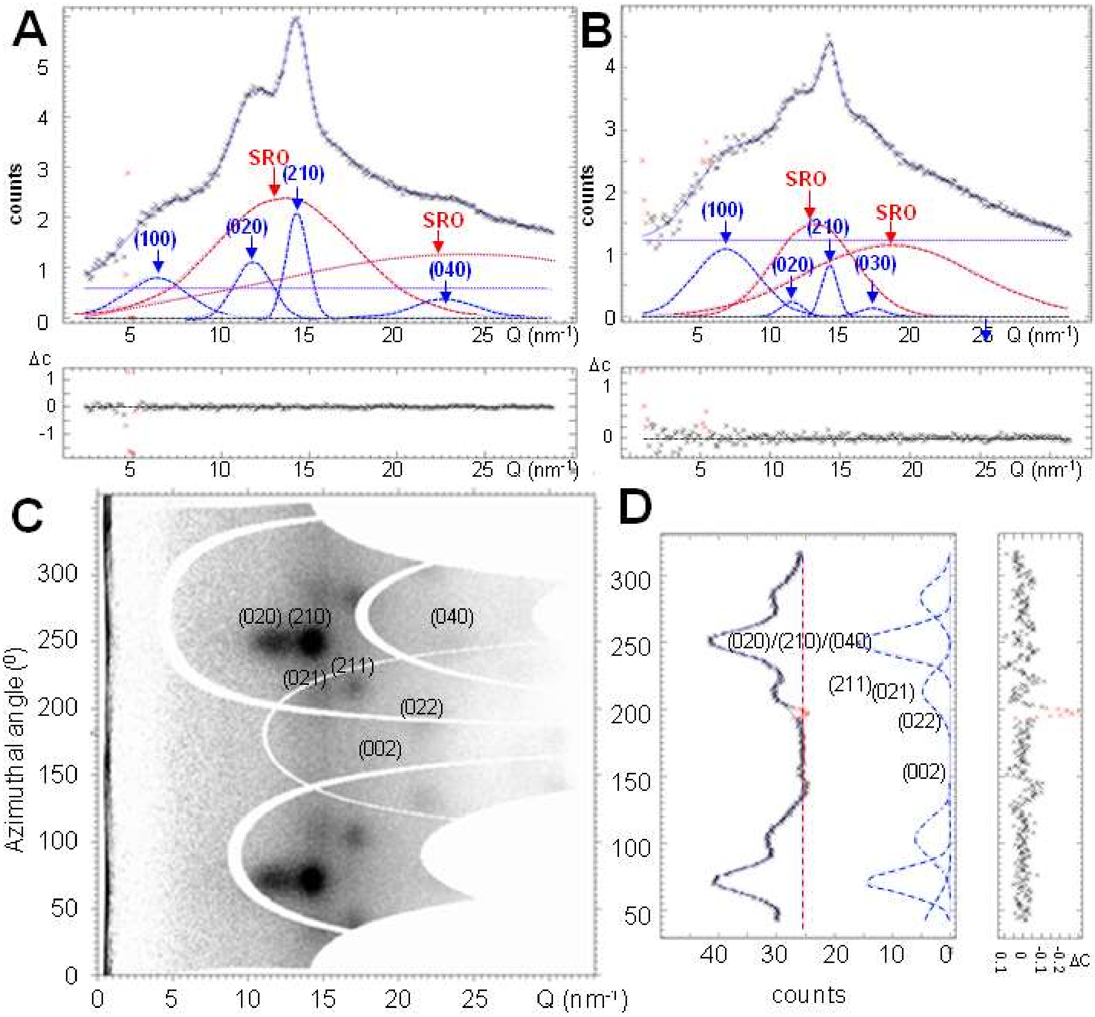


**SM Fig. 1** (A) Equatorial intensity profile of radial thread simulated by Gaussians for Bragg ( in blue) and SRO (in red) peaks and a 0-order polynomial for random residual scattering. Miller's indices associated with the Bragg peaks of the β-sheet nanodomains correspond to the poly(L-alanine) lattice. $\Delta C = C_{exp} - C_{sim}$ is the differential intensity. (B) Equatorial intensity profile of hub-spiral simulated by Gaussians as for (A). Bragg peaks indexed for the poly(L-alanine) lattice. (C) Cake-regrouped 2D MaS pattern. (D) 1D azimuthal intensity distribution of pattern in (C) obtained by integrating along Q-axis. The azimuthal intensity distribution was fitted by Gaussians Bragg peaks. SRO scattering is contained in the random background fitted by a 0-order polynomial. "Red" pixels in (A-C) due to non-sensitive detector areas were masked for the fits. $Q=2\pi d^{-1}=4\pi \sin\Theta \lambda^{-1}$ where d is the lattice spacing, Θ the Bragg angle and λ the wavelength.

The scattering background was reduced by a He-path between slit system and beamstop. The sample was aligned by an Olympus optical microscope in the SR focal spot and mesh-scanned normal to the beam direction along orthogonal horizontal and vertical axes. Scanning

microXRD was performed by an M-810 6-Axis Hexapod (PI) with ±500 nm repeatability and scanning nanoXRD by a Hera 2D piezostage (PI) with ±10 nm repeatability. X-ray radiation damage due to radicals propagation into unexposed mesh-points next to an exposed mesh-point was limited for scanning nanoXRD by choosing a step-width of 0.5µm (hxv)[1]. Micro-/nanoXRD patterns were collected at each step by an EIGER 4M pixel detector (DECTRIS) with 2070 x 2167 pixels of 75µm x 75µm area each, single photon counting capability and no readout noise. The position of the beam was displaced from the centre of the detector to obtain access to larger Q-values for part of the pattern.

**Data reduction and display.** FIT2D was used for analysis and display of selected patterns[4]. The composite diffraction image (called "composite image"; Abbr.: CI) obtained from the patterns of a mesh-scan is proportional to an electron (or mass) density projection at the hierarchical scale determined by the selected angular range of the "pixels" (patterns). SAXS-CIs based on pixels covering the SAXS and WAXS-CIs covering the SAXS/WAXS range were generated from the sequence of patterns obtained in a mesh-scan. Background scattering obtained close to the sample was subtracted. For the weakest scattering features from the $am_2$ fiber we analyzed the raw detector data.

The equatorial intensity profile obtained at the marked position of a radial fiber in a microXRD WAXS-CI (SM Fig. 2E) is shown in SM Fig. 1A. The corresponding 2D pattern is shown in SM Fig. 2A and the cake-regrouped pattern in SM Fig. 1C. The Q-scale is defined as: $Q=2\pi\sin\Theta\lambda^{-1}=2\pi d^{-1}$ where Θ: Bragg angle, λ: wavelength, d: lattice spacing. The profile was fitted by six Gaussians for Bragg peaks and diffuse scattering (called "SRO" for "short-range order scattering") as well as a 0-order polynomial for residual scattering[1]. Intensity profiles and peak fit parameters were also plotted using the Origin® software.

**WAXS analysis.** The particle size ($L_{[hkl]}$) is determined from the full-width-at-half-maximum (fwhm) of the Gaussian fitted to a (hkl) Bragg peak according to Scherrer's equation[5]. It corresponds to a lower limit in particle size as additional possible broadening effects (e.g. strain) are not considered. The particle size determination for MaS fibers has been reported in[1].

The "orientation distribution" ($f_c$) of crystalline β-sheet nanodomains along the fiber axis was determined in terms of Herman's orientation function[6]:

$$f_c=(3<\cos^2\phi_c>-1)/2 \qquad \text{SM Eq. 1}$$

varying between 0 (random orientation) and 1 (perfect alignment). $f_c$ was derived from the azimuthal width $\phi_{020/210}$ of the equatorial (020)/(210) reflections via[7,8]:

$<\cos^2\phi_c>=1-0.8<\cos^2\Phi_{020}>-1.2<\cos^2\Phi_{210}>$ SM Eq. 2

where $\Phi_{020/210}=90-\phi_{020/210}$.[1] The azimuthal peak profiles were determined by fitting the radially integrated (020) and (210) peaks by a narrow Gaussian for the (hk0) peaks, a broad Gaussian covering the (hk1) layer-line peaks and a 0-order polynomial for residual scattering[9]. For the amorphous fibers, the azimuthal width of the equatorial streak was extrapolated to a constant value at the largest accessible Q-value to separate orientation distribution from particle size[10-12].

The crystallinity $X$ (%) of silk fibers is defined as:

$$X = \frac{\Sigma I_B}{\Sigma I_B + \Sigma I_{SRO}} x100 \quad \text{SM Eq. 3}$$

$\Sigma I_B$ is the sum of the integrated Bragg peak intensities and $\Sigma I_{SRO}$ the sum of the SRO intensities. For the MaS fibers, Bragg intensities were obtained from the azimuthal intensity profiles via the full-width at half maximum (fwhm) of the Gaussian peak profiles ($\sigma_{Intensity}$x2.354) and the SRO intensity ($I_{SRO}$) from a box function of $I_{max}$*angular range ($^0$) (SM Fig. 1D). For a different approach in determining the contributions to SM Eq. 3 see:[7]. The quasi-linear difference pattern of observed minus simulated intensities ($\Delta c=\Delta I_{obs-sim}$) suggests that the assumption of an absence of a preferred SRO orientation is a good approximation. $X$-values are more qualitative for lower-crystallinity fibers such as stabilimentum threads in view of the difficulty in separating crystalline from SRO scattering[13].

**SAXS analysis.** The meridional SAXS peak of MaS fibers is attributed to nanofibrils composed of crystalline nanodomains stacks separated by less ordered protein, providing a density modulation along the nanofibrillar axis[1]. Scattering is Bragg-type although deviations from an ideal stacking period imply that only one or sometimes two orders are observed[1,11]. For more crystalline (44 %) bagworm silk several meridional peaks are observed[14].

The equatorial SAXS streak, which is also observed for amorphous silk fibers, is attributed to the reciprocal space transform of bulk fibrillar objects. The intensity of the streak can be formulated as product of a form factor term *P(Q)* and density contrast between fibrils and surrounding matrix $\Delta\rho$:

$$I(Q) = k' P(Q)\Delta\rho \quad \text{SM Eq. 4}$$

where $k'$ is a scaling factor[11,12]. The (scattering) density contrast and therefore the intensity of the streak can be modified and quantitatively analyzed by H/D exchange using small-angle neutron scattering (SANS) as shown for *Nephila* MaS silk[12]. The extent of deuteration is

known as the H/D exchange rate is very slow for the crystalline polyalanine-rich β-sheet nanodomains but fast for the disordered polyglycine-rich polypeptidic chains in the matrix surrounding the nanofibrils and the chains connecting the nanodomains within the nanofibrils. The $\Delta\rho$ term of the SAXS streak intensity can be modified by changing the density of the matrix surrounding the nanofibrils through hydration[15]. The scattering contribution from the mesofibrillar MaS skin-layer[1] to the equatorial streak is weak and cannot be separated from the SAXS streak for scattering experiments on fiber bundles using larger X-ray beams.

Simulation of fibrillar scattering was based on the assumption of uncorrelated cylinders with radius $r$ resulting in a modulated intensity decay: [12,16]

$$P(Q) = c\frac{r^4}{Q}\left(\frac{2J_1(Qr)_1}{Qr}\right)^2 \qquad \text{SM Eq. 5}$$

where $J_1$ corresponds to the Bessel function of first kind and first order and c is a scaling constant. The instrumental resolution of the micro-/nanoSAXS setups limits the observation of modulated intensity decay to cylindrical diameters ($d_c$) of approximately 100 nm. This dimension correspond to mesoscale bundles of aggregated nanofibrils observed for bagworm silk[14]. A cylindrical diameter can also be determined from the slope (m) of the $J_1$ orders versus Q via $d_c=2\pi/m$.[17] The modulation period becomes too large for nanofibrils with $d_c<10$ nm. An approximate $d_c$-value can, however, be derived by determining the Guinier radius of gyration $R_G$ from the slope of the equatorial streak intensity via a $\ln(I*Q)/Q^2$ regression line fit providing $d_c=R_G*8^{0.5}$.[11] If observable, one can also deduce the diameter of close-packed nanofibrils from an equatorial SAXS correlation peak.

### Orb-web features in composite images

**Radial thread.** A microXRD pattern from one of the of 4 parallel strands in the SAXS&WAXS-CIs (Fig. 2A, SM Fig. 2G) shows equatorial poly(L-alanine) lattice Bragg peaks from the crystalline β-sheet nanodomains of MaS fibers[1,7,18], SRO scattering from the disordered polypeptidic matrix and a meridional SAXS correlation peak (also called “long period”) attributed to nanofibrils[1,11] (SM Fig. 2A,B). We determined a crystallinity of ~14 % according to SM Eq. 3. The equatorial intensity profile (SM Fig. 1A) agrees also to MaS fibers’ intensity profile[1], set aside a more intense peak at the (100) position which would imply a breakdown of the $2_1$ symmetry along the a-axis of the poly(L-alanine) lattice[18].

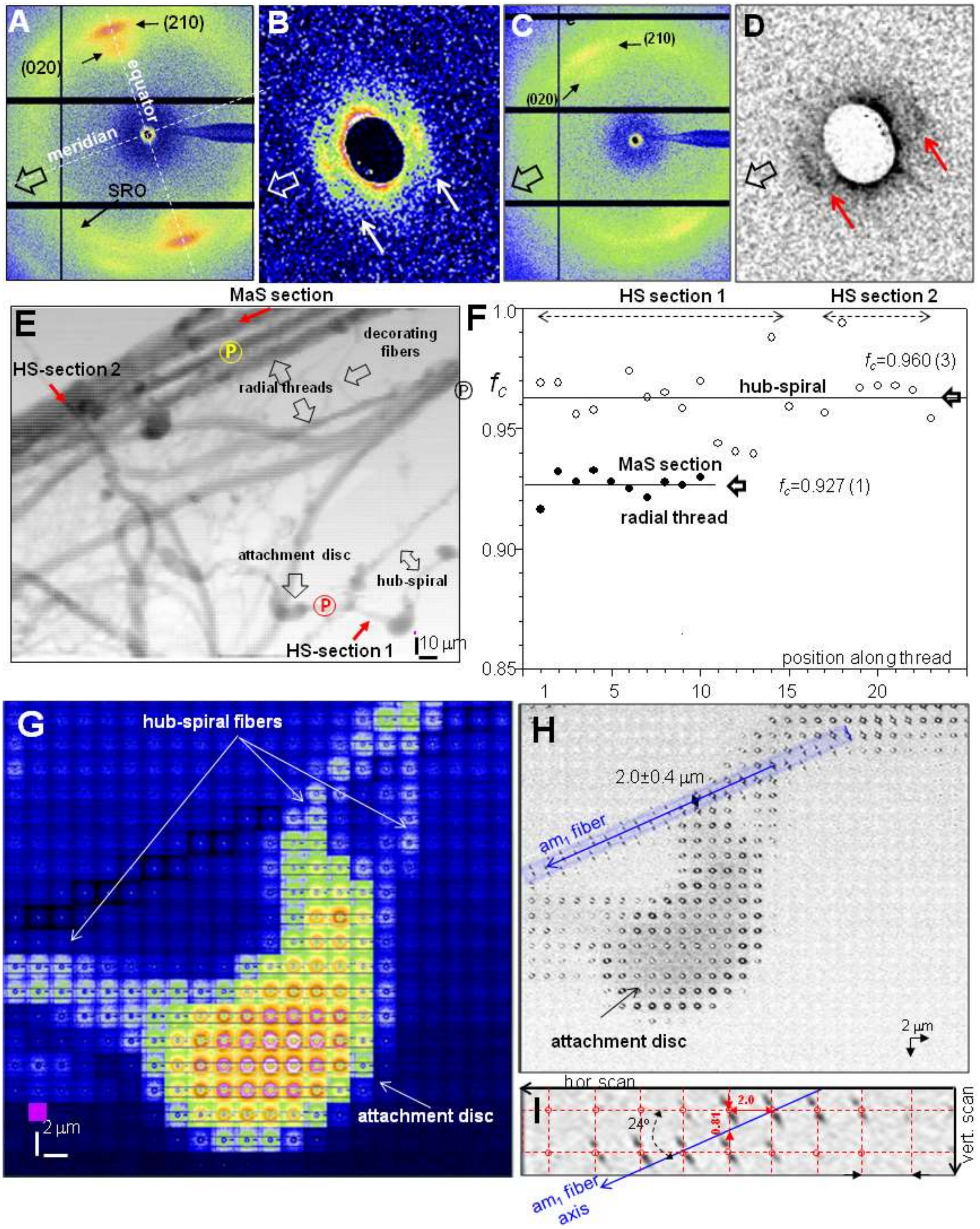


SM Fig. 2 (A,B) WAXS patterns from radial thread at yellow Ⓟ in (E) with poly(L-alanine) reflections and SRO powder ring; SAXS pattern with meridional peaks (white arrows). (C,D) SAXS/WAXS pattern of hub-spiral at red Ⓟ in (E). Open arrows along fiber axis. (E) WAXS-CI; red arrows indicate sections sampled along radial thread (MaS section) and hub-spiral (HS sections). (F) Hermann's orientation function values ($f_c$) along radii thread and hub-spiral sections. Horizontal lines correspond to linear regression fits to the $f_c$ values. (G) Zoom of WAXS-CI hub-spiral thread traversing an attachment disc. The beam size is schematically shown as a pink square. (H) Zoom of SAXS-CI with $am_1$ fibers projection (in blue) defined by SAXS streaks. The diameter of the fiber is estimated to be 2.0 ±0.4 µm (see below). (I) Two scan-lines across $am_1$ fiber. NanoXRD patterns were collected at positions of the filled circles; each 2.0 µm horizontal scan-step implies a vertical beam displacement across the fiber by 0.81 µm allowing increasing the number of horizontal sampling points across the fiber by about factor 2.5. The ±0.4 µm error in diameter is due to the size of horizontal scan-increments, limiting the accuracy in determining the range of horizontal streak scattering.

We do not, however, observe an azimuthally well-defined peak at this position in the diffraction pattern (SM Fig. 2C) suggesting diffuse, SRO-type scattering from an undefined silk fraction. The nanodomains axial orientation distribution along the radial threads section of $f_c$=0.927(1) (SM Figs. 2E,F) agrees to $f_c$-values from *A. bruennichi's* bridge-thread[1].

**Hub-spiral thread: MiS or MaS**? A zoom of the WAXS-CI reveals the two fibers of a thread traversing an attachment disc which has lost its connection to the radial fibers (SM Fig. 2G, SM Fig. 2E). The absence of glue droplets excludes a capture-thread and supports a hub-spiral thread[19]. The ~4 µm diameter fibers show the poly(L-alanine) Bragg peaks of MaS fibers including a meridional SAXS peak (SM Figs. 1B; 2C,D). The peak at the (100) position in the equatorial intensity profile (SM Fig. 1B) does, however, not show up as an azimuthally defined peak in the diffraction pattern (SM Fig. 2C) and is probably also due to an undefined silk material as for the radial fibers. We observe stronger SRO scattering as compared to the radial fibers, reducing the crystallinity to $X$~10 %. The hub-spiral has been attributed to MiS fibers[20] containing β-sheet protein motifs[21]. An alternative proposal based on morphological and mechanical properties attributes the hub-spiral rather to MaS fibers[22]. Attempts differentiating MiS from MaS fibers from forced-silked *Nephila* and *Argiope* spiders by their crystallinity, particle size or orientation function ($f_c$) values have not been successful[23]. Given the difficulty of separating SRO from Bragg scattering for the hub-spiral fiber (SM Fig. 1B) we analyzed parameters derived from the Bragg peaks such as unit cell metrics via Bragg peak positions, particle size or orientation distribution (SM Eq. 1,2). Bragg peak positions and nanodomains size values (SI Table 1) of hub spiral and radial thread are again quite similar.

SI Table 1. Particle size ($L_{hk0}$) based on Scherrer's formula[5] along [hk0] lattice directions, normal to the fiber axis (c) of the poly(L-alanine) structure[18].

| | $L_{[100]}$ (nm) | $L_{[010]}$ (nm) | $L_{[210]}$ (nm) |
|---|---|---|---|
| hub-spiral | 1.3 | 2.2 | 3.9 |
| radial thread | 1.3 | 2.7 | 3.8 |

$f_c$ values from various spider species show a too high variability for a systematic comparison[1,7-9,24,25,26]. In the current case all fibers probed and analyzed were, however, from the same orb-web and sampling along fiber sections was performed. We also followed a common data reduction protocol including background treatment, fitting the 360$^0$ azimuthal profile by Gaussians for the two symmetry-equivalent Bragg peaks as well as two broader Gaussians covering the 1$^{st}$ layer-line peaks[9]. Indeed, we determine a significantly higher $f_c$=0.960(3) value for the hub-spiral thread as compared to the radial thread ($f_c$=0.927(1); SM

Fig. 2F). The drawing speed is known influencing the $f_c$-value for *Nephila* MaS fibers[24]. The difference in $f_c$-values shown in SM Fig. 2F is, however, too large to be explained by different MaS drawing speeds for hub-spiral and radial threads during orb-web building with drawing speeds of <10 mms$^{-1}$. In conclusion, our data show that hub-spiral fibers have the same composite nature of β-sheet nanodomains in a disordered matrix as radial thread or bridge-thread. The only feature pointing to two different types of fibers are differences in $f_c$-values. Within the frame of an affine deformation model proposed for MaS fibers[24] this could be due to differences in interactions of anisotropic β-sheet nanodomains with the elastic matrix. The origin could be due to different nanodomains volume fractions (i.e. different crystallinities) of MaS and MiS fibers.

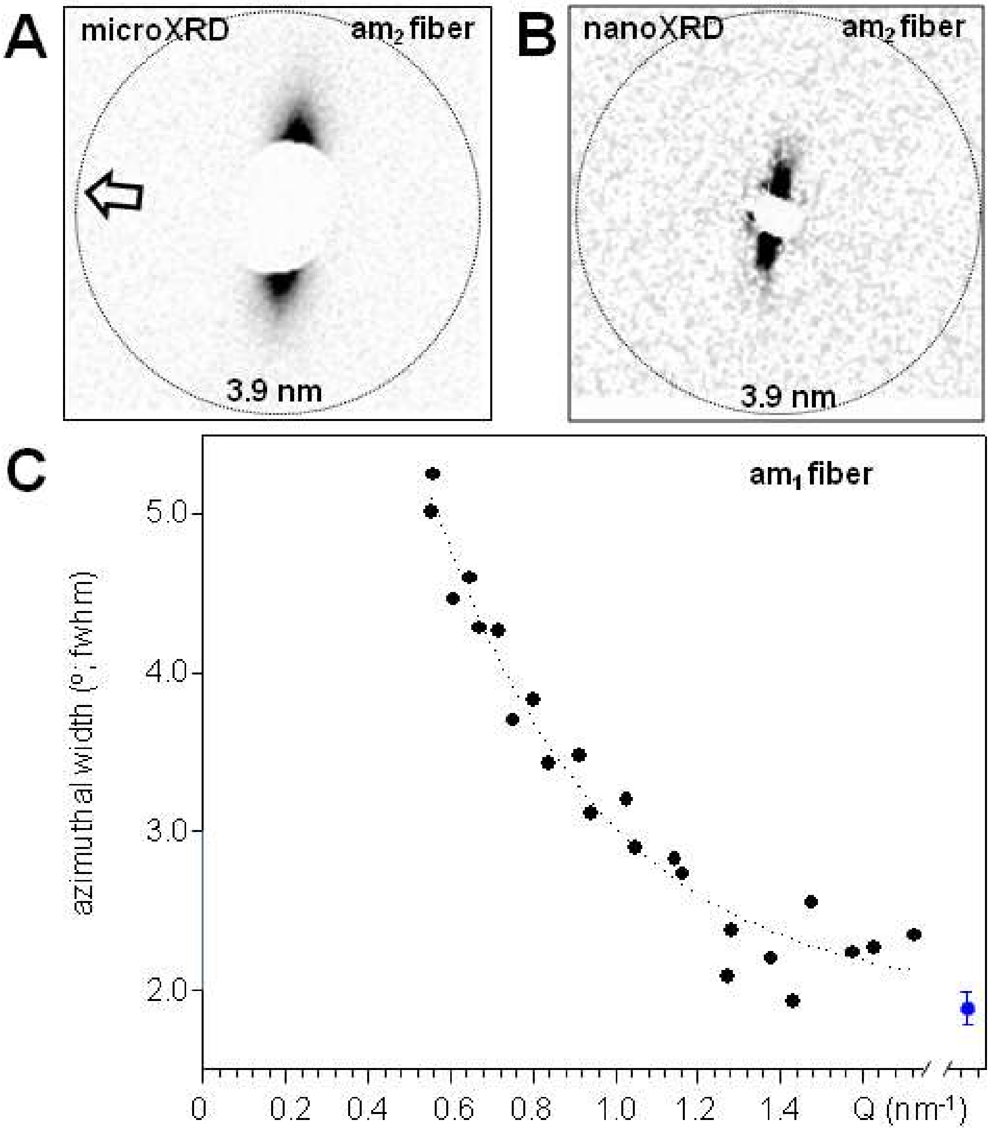


SM Fig. 3 (A) SAXS streak of am$_2$ fiber by microXRD; open arrow along fiber axis; azimuthal width at Q=1.2 nm$^{-1}$: 18° fwhm. (B) SAXS streak of am$_2$ fiber by nanoXRD; average of whole scan-line of CI (SM Fig. 6B,C); azimuthal width at Q=1.2 nm$^{-1}$: 15° fwhm; azimuthal width of single streak: 8° fwhm. (C) Q-dependant azimuthal width of am$_1$ fibers SAXS streak. A 2$^{nd}$ order exponential decay has been fitted. The blue data point of 1.9$^0$ (±0.3) width provides $f_c$=0.998(1).

**Stabilamentum fibers** show a low crystallinity of <5% observed also for other aciniform silk fibers[13,27]. The mesh of fine fibers is revealed by dominant SRO scattering in the WAXS-CI suggesting strong hydrogen-bonding mediated chain interactions contributing to mechanical features (Fig. 2A, SM Fig. 2E). The fiber contours are also revealed in the microXRD SAXS-CI (Fig. 2B). NanoXRD SAXS-CIs reveal single stabilimenta fibers with mesofibrillar features at the rim[13].

**Scanning nanoXRD of am$_2$ fiber**

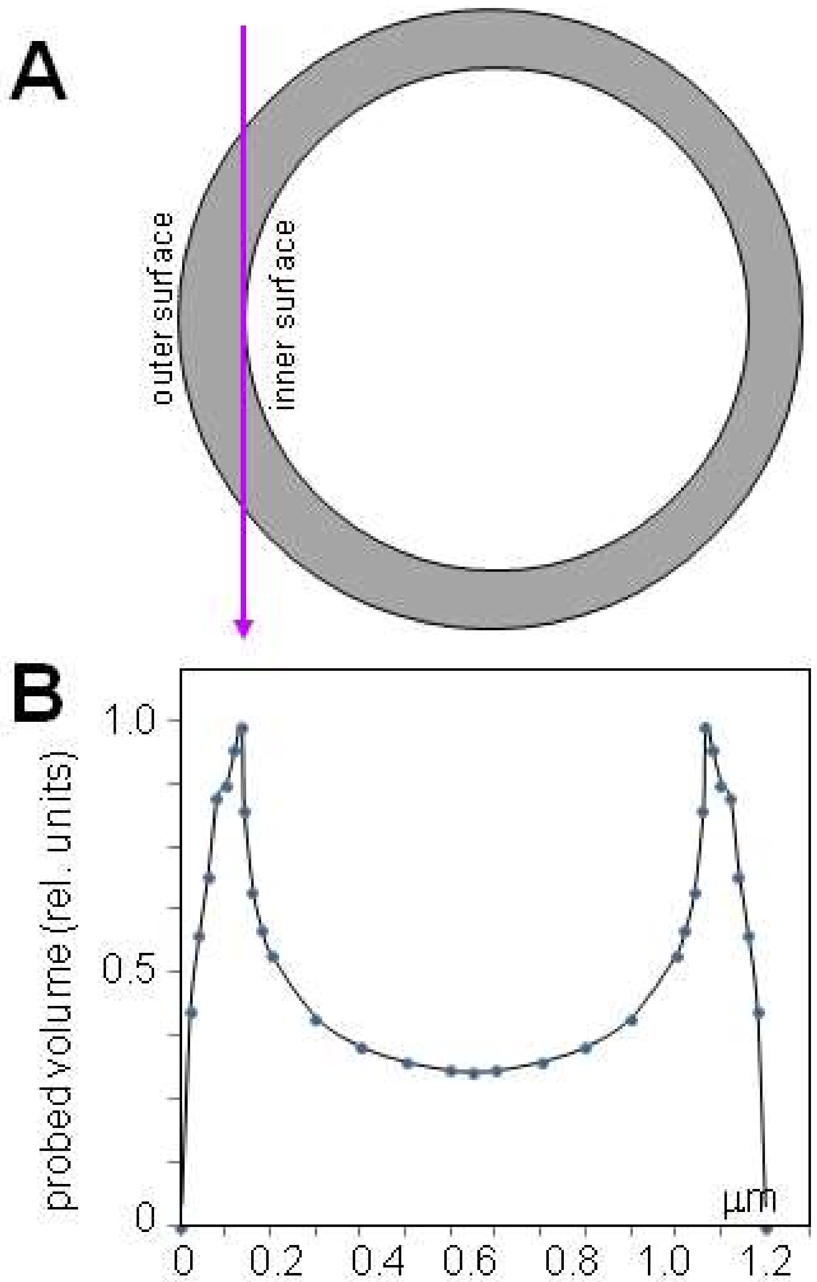


SM Fig. 4 Schematic design of scanning a 1.3 µm diameter hollow fiber with a 0.13 µm thick skin-layer through a pencil-like X-ray beam. The probed volume is assumed to be proportional to the length of the beam intersecting the solid part of the fiber. (A) Cross-section of the hollow fiber. The beam (in pink) is shown probing the fiber at the inner surface. (B) Probed volume when moving fiber horizontally through the beam.

**Banded features on MaS fibers**

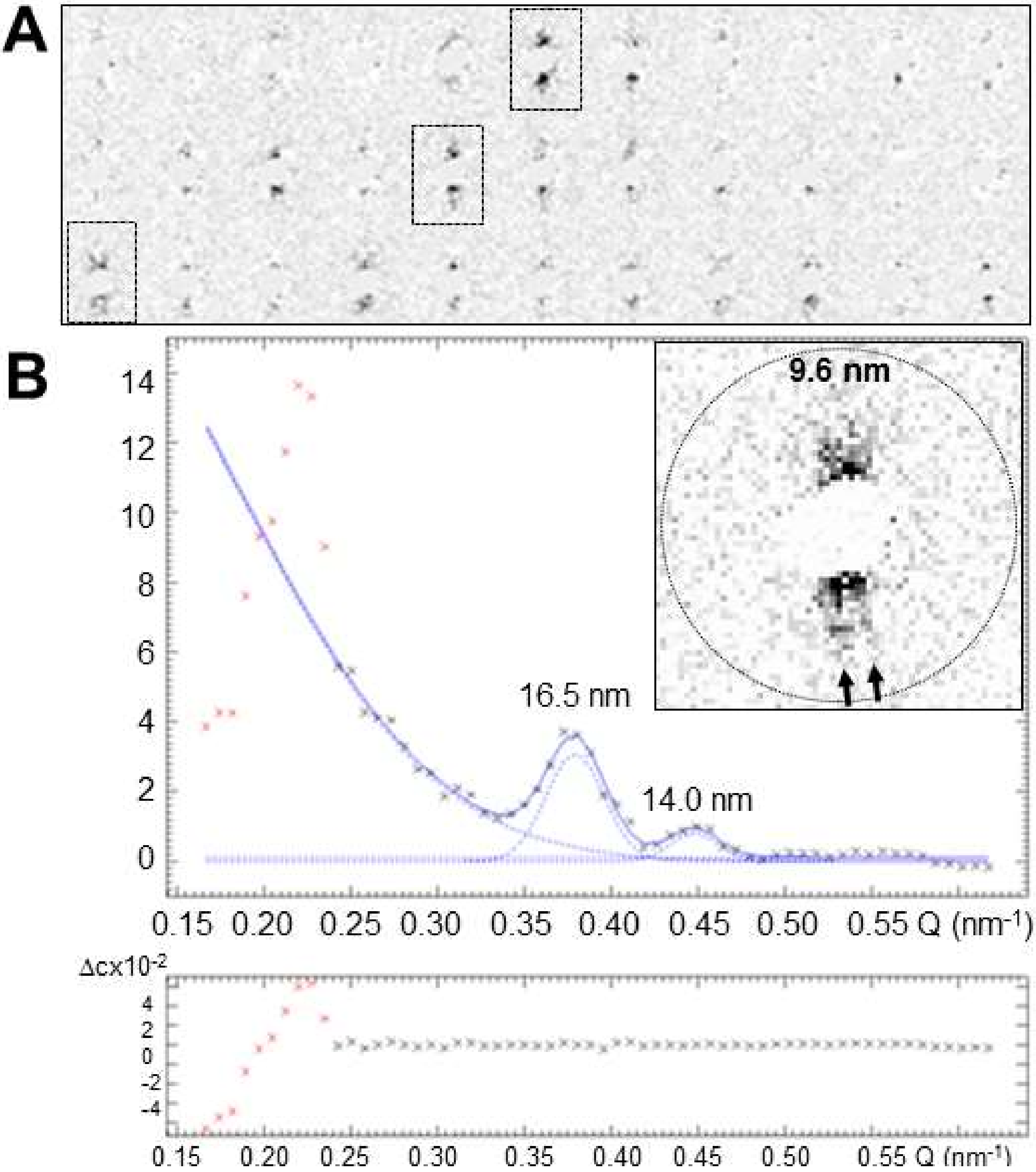


SM Fig. 5 (A) Zoom into nanoXRD SAXS-CI (Fig. 6A) centered on streak from a single band. MaS scattering has been subtracted. Three streaks indicated by dotted rectangles were averaged. (B) Inset: averaged streak from (A). Two sub-streak orientations are indicated by arrows. Upper radial intensity profile: azimuthal average of lower streak in inset. The profile was fitted by 3 Gaussians for peaks and continuous intensity decay; 0-order polynomial for the random background (blue curves). The red data points were not included in the fits. Lower intensity profile: difference of observed data minus simulated curve.

The SAXS-CI reveals a banded morphology (Fig. 6A) due to streaks from fine fibers at the MaS surface (Figs. 3A, 6C). We selected an area of a single band in the SAXS-CI (Fig. 6A; dashed rectangular area) corresponding to a composite of streaks from MaS nanofibrils[1] and weaker streak from the fine fibers. We subtracted the MaS scattering from each pattern by a pattern outside the area of enhanced scattering from the band. This allows maintaining

approximately equal scattering volumes of the patterns resulting in differential patterns with enhanced streak scattering from the surface fibers. In view of the rather small lateral extension of the bands and the potential overlap with neighboring bands including gaps, we averaged three of the strongest patterns to maximize scattering from the single band (SM Fig. 5A). The enhanced SAXS streak (SM Fig. 5B, inset) is split, suggesting two fibrillar orientations of about $15^0$ azimuthal angular differences. The radial intensity profile is modulated suggesting that the surface fibrils are composed of mesofibrils while MaS streaks from the core show a continuous intensity decay due to <10 nm nanofibrils[1].

**Fibrillar skin of am₂ fibers**

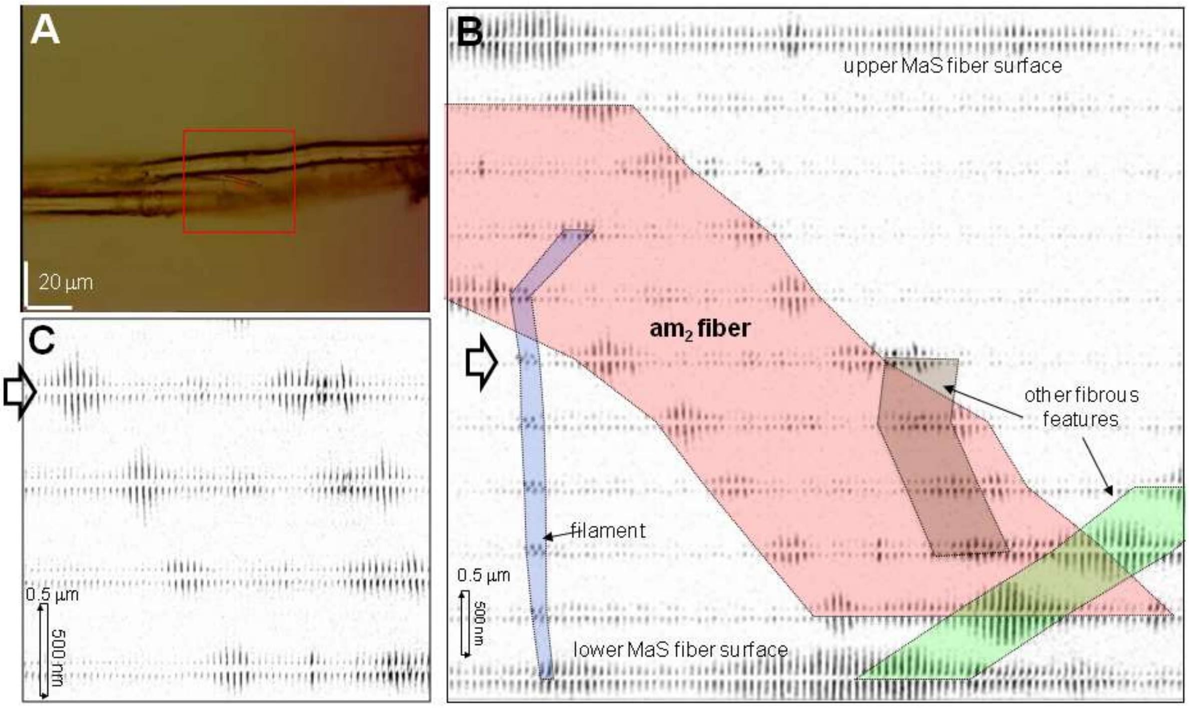


SM Fig. 6 (A) Optical microscopy of MaS thread and bridging $am_2$ fiber. The red rectangle indicates the area scanned by nanoXRD. (B) SAXS-CI with pixels limited to equatorial streaks revealing the lower and upper MaS fibers surface and several fibrous features in the gap between the two MaS fibers. The outline of the $am_2$ fiber (in red) is defined by the outer streaks of the group of streaks in each horizontal line. The streaks allow tracing three further fibrillar features which are distinguished by different colours. The continuation of the $am_2$ fiber into the projection of the MaS fibers (Fig. 6A) is not shown. (C) Zoom of $am_2$ fiber in (B). Aspect ratio ~8:1 in (B,C) for better visualization of the spatial distribution of the fibrillar features. The separation of a $2^{nd}$ group of streaks can be seen at the right edge of the $am_2$ fiber for the line depicted by an open arrow,

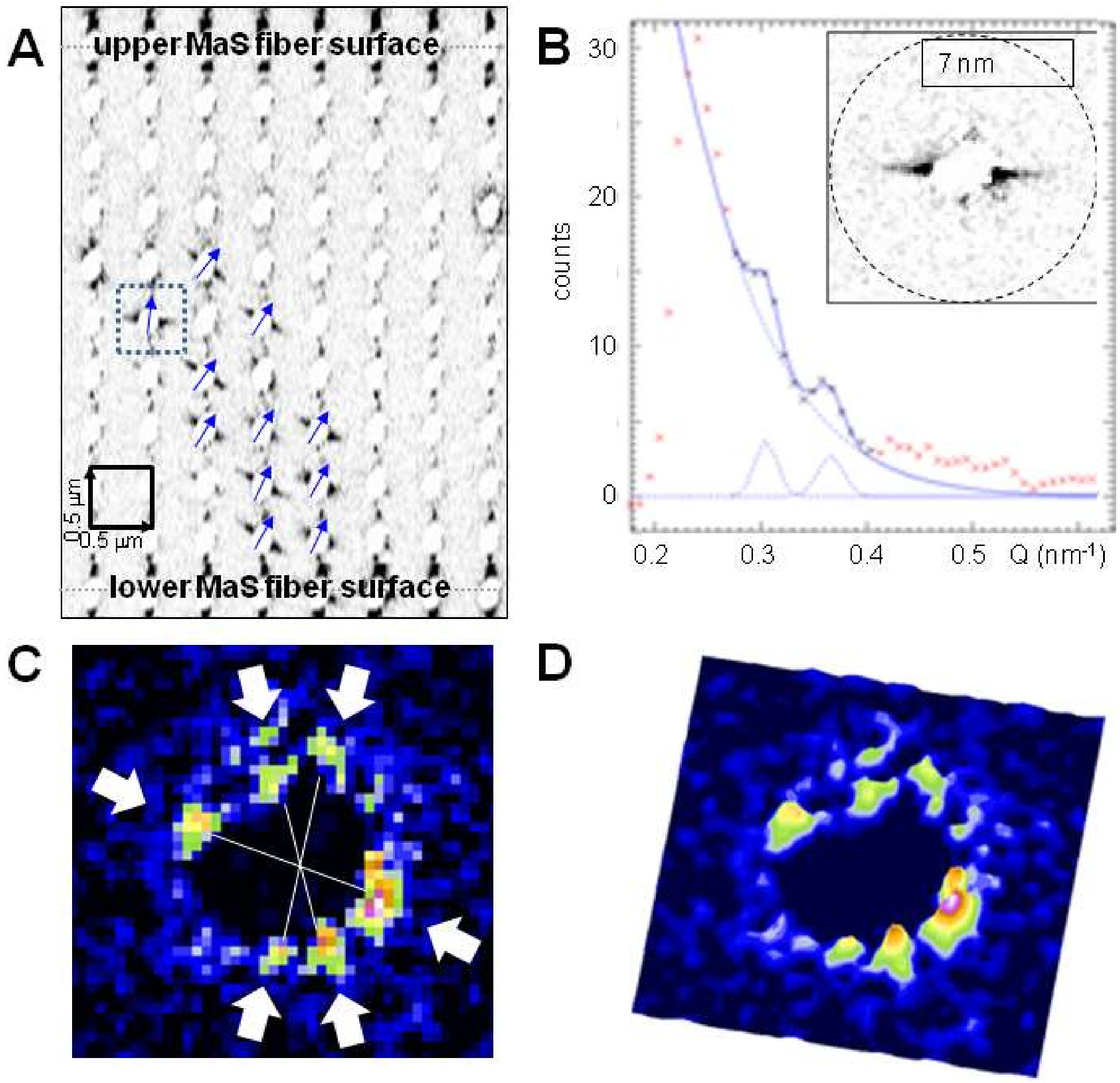


SM FIG. 7 (A) SAXS-CI revealing scattering from filament connecting $am_2$ fiber to lower MaS surface. The pixels cover the more or less horizontal streaks from the filament. Orthogonal local filament directions are indicated by blue arrows. (B) Radial intensity profile of streak from dotted square in (A). A zoom of the streak is shown as inset. Two peaks were fitted to the curve on a broad Gaussian background. The red data points were masked for the fit. (C) SAXS pattern from about the middle of the $am_2$ fiber; three weak streaks are indicated by arrows. The peak at the edge of the beamstop corresponds to about 16.7 nm. (D) Pseudo 3D plot of C).

**Intensity profile of am2 fibers' equatorial streak**

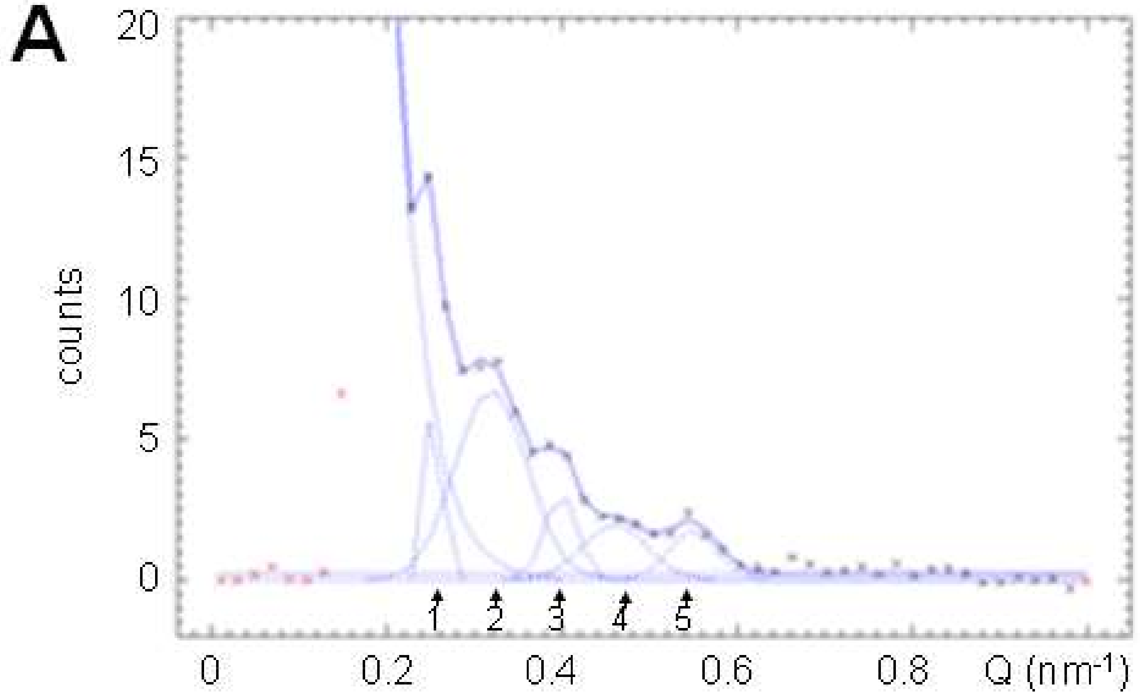


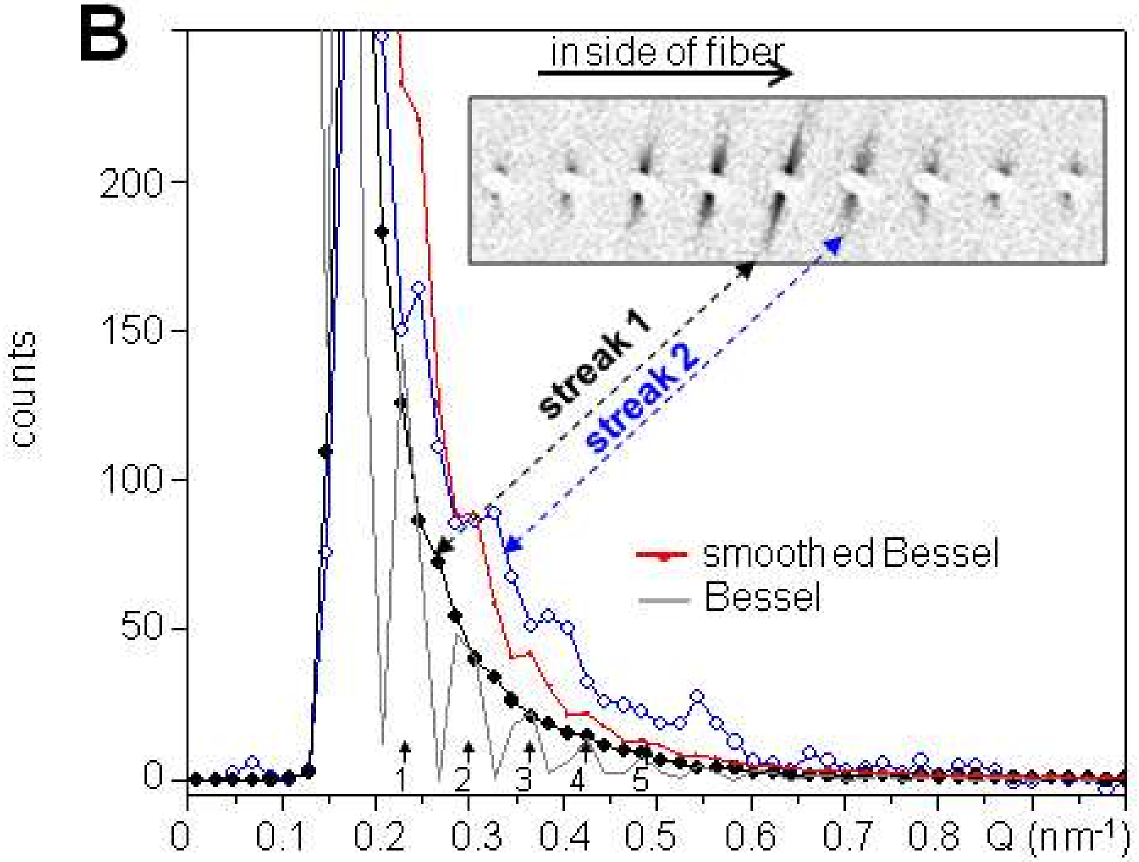


SM Fig. 8 (A) Intensity profile of modulated SAXS streak 2 fitted by 5 Gaussians for the peaks and a broad Gaussian for the nonlinear background. (B) Intensity profile of streaks 1/2 scaled to the strongest intensity of streak 1 (in black). Streak 2 (in blue) was fitted by a 1st order Bessel function for cylinders with 100 nm diameter). The Bessel function is shown unsmoothed (in grey) and smoothed (in red) by averaging neighbouring points.

## PG-II nanocrystallites attached to am$_2$ fiber

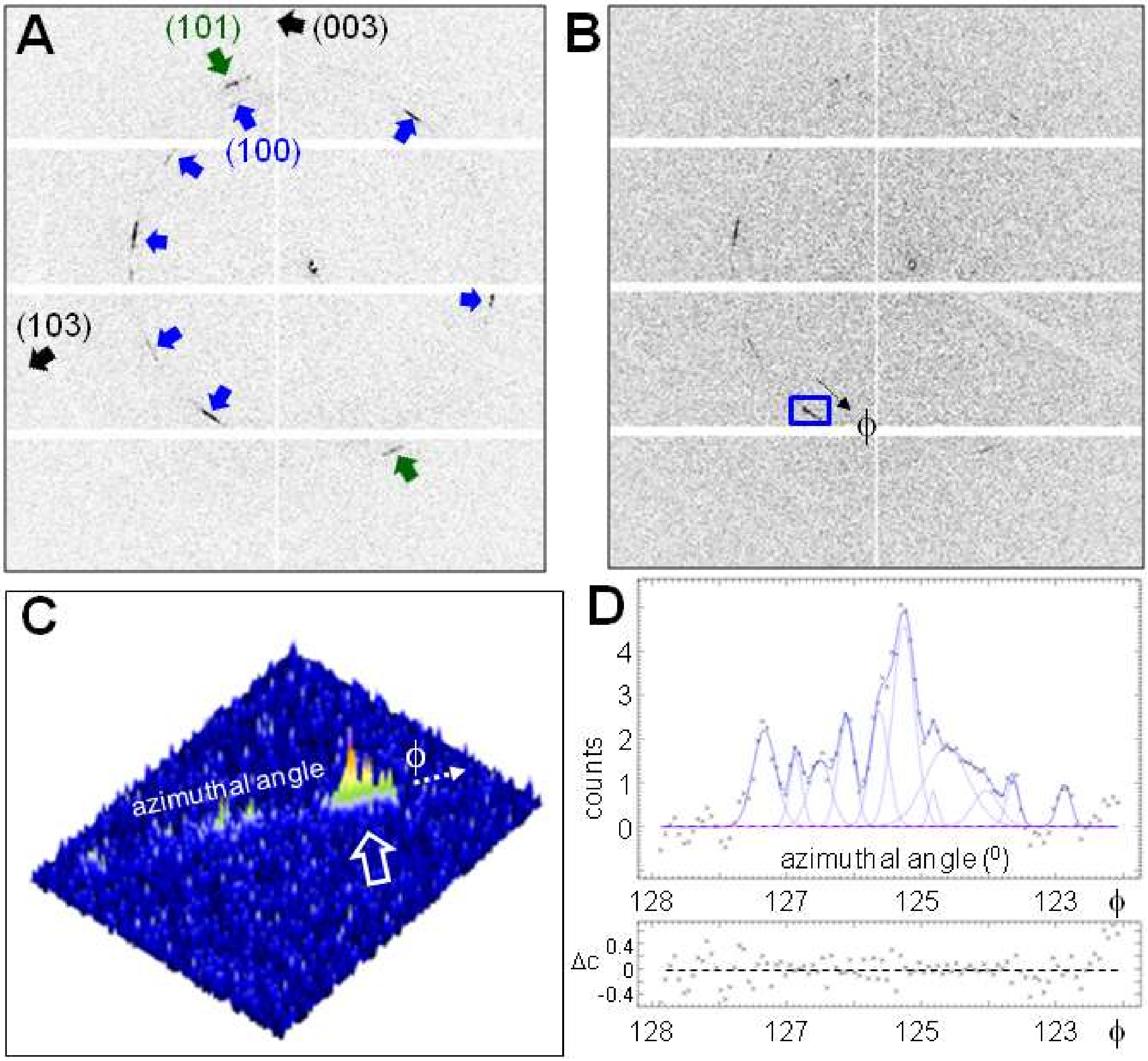


SM Fig. 9 (A) Average of 13 textured patterns of nanocrystallites attached to am$_2$ fiber (Fig. 4E). The Bragg peaks are indexed for the PG-II lattice[28]. The angular distribution of the strong (100) reflections (blue arrows) resembles a pseudo-hexagonal texture. Weak PG-II (003)/(103) peaks are observed at two positions. (B) Single pattern. (C) Pseudo 3D plot of cluster of (100) peaks from blue rectangle in (B). (D) Azimuthal ($\phi$) intensity profile of cluster of PG-II (100) peaks indicated by open arrow in (C) fitted by 11 Gaussians with 0.05-0.2$^0$ fwhm.

**Rupture zones of detached am2 fiber on MaS thread**

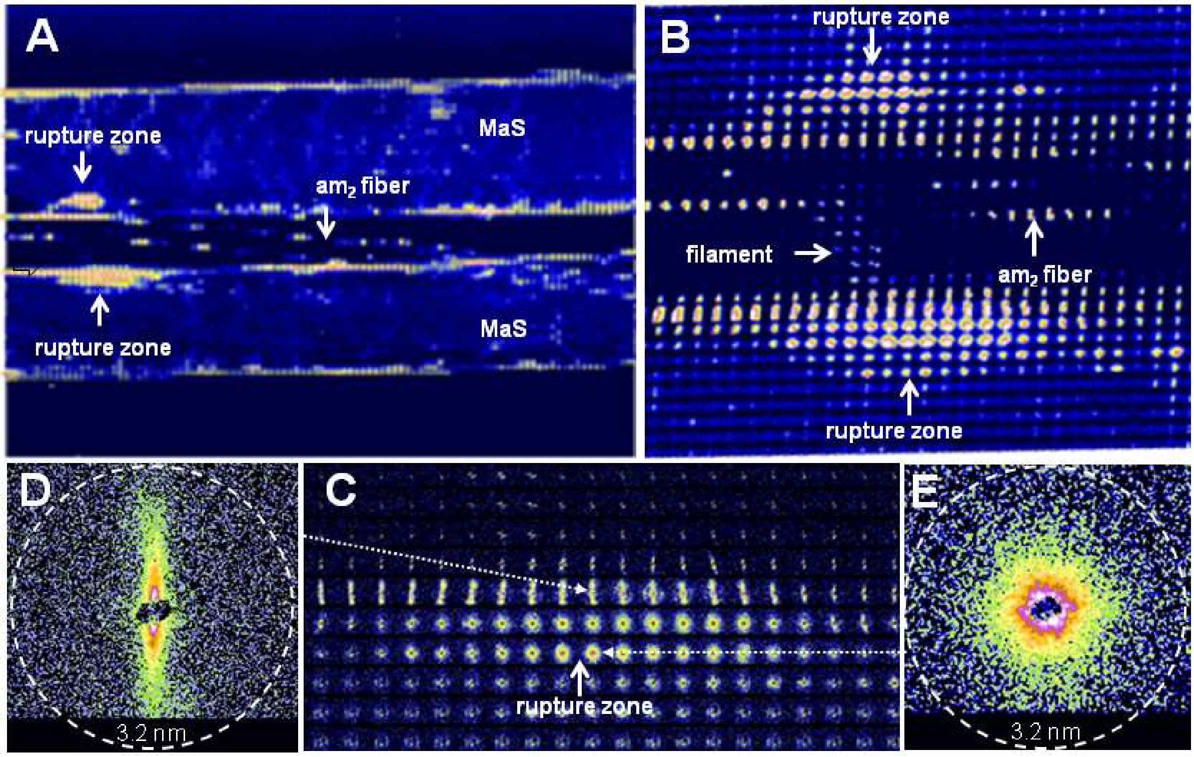


SM Fig.10 (A) CI of MaS thread with bridging $am_2$ fiber based on pixels covering the equatorial SAXS streak (16x binned to enhance weak features). (B) Pseudo 3D zoom of rupture zones revealing a filament connecting the $am_2$ fiber to the MaS fiber surface. (C) Zoom into CI of lower rupture zone (log intensity; not binned) showing l SAXS streaks at the edge the MaS fiber skin (D) and random SAXS scattering from the rupture zone (E).

## References


1 Riekel, C., Burghammer, M., Ferrero, C., Dane, T. & Rosenthal, M. Nanoscale Structural Features in Major Ampullate Spider Silk. *Biomacromolecules* **18**, 231-241, doi: 10.1021/acs.biomac.6b01537 (2017).

2 Riekel, C., Burghammer, M., Davies, R., Gebhardt, R. & Popov, D. in *Applications of Synchrotron Light to Scattering and Diffraction in Materials* Vol. 776 *Lecture Notes in Physics* (eds T.A. Ezquerra, M. Garcia-Gutierrez, A. Nogales, & M. Gomez) 91-104 (Springer, 2009).

3 Lengeler, B. *et al.* Parabolic Refractive X-Ray Lenses: a Breakthrough in X-Ray Optics. *Nuclear Instrum. Meth. A* **467-468**, 944-950, doi: 10.1016/S0168-9002(01)00531-9 (2001).

4 Hammersley, A. P. FIT2D: a Multi-Purpose Data Reduction, Analysis and Visualization Program. *J. Appl. Cryst.* **49**, 646-652, doi: 10.1107/ S1600576716000455 (2016).

5 Klug, H. P. & Alexander, L. E. *X-ray Diffraction Procedures for Polycrystalline and Amorphous Materials*. 2nd edn, (Wiley Interscience, 1974).

6 Stein, R. S. & Wilkes, G. L. in *Structure and Properties of Oriented Polymers Materials Science Series* (ed I.M. Ward) 57-145 (Applied Science Published Ltd, 1975).

7 Grubb, D. T. & Jelinski, L. W. Fiber Morphology of Spider Silk: The Effects of Tensile Deformation. *Macromolecules* **30**, 2860-2867, doi: 10.1021/ma961293c (1997).

8 Riekel, C. *et al.* Aspects of X-ray Diffraction on Single Spider Fibers. *Int. J. Biol. Macrom.* **24**, 179-186, doi: 10.1016/s0141-8130(98)00084-1 (1999).

9 Du, N. *et al.* Design of Superior Spider Silk: From Nanostructure to Mechanical Properties. *Biophys J* **91**, 4528–4535, doi: 10.1529/biophysj.106.089144 (2006).

10 Grubb, D. T., Prasad, K. & Adams, W. W. Small-Angle X-Ray Diffraction of Kevlar using Synchrotron Radiation. *Polymer* **32**, 1167-1172, doi: 10.1016/0032-3861(91)90217-7 (1991).

11 Yang, Z., Grubb, D. T. & Jelinski, L. W. Small-Angle X-ray Scattering of Spider Dragline Silk. *Macromolecules* **30**, 8254 - 8261, doi: 10.1021/ma970548z (1997).

12 Sapede, D. *et al.* Nanofibrillar Structure and Molecular Mobility in Spider Dragline Silk. *Macromolecules* **38**, 8447-8453, doi: 10.1021/ma0507995 (2005).

13 Riekel, C., Burghammer, M. & Rosenthal, M. Nanoscale X-Ray Diffraction of Silk Fibers. *Frontiers in Materials* **6**, 315, doi: 10.3389/fmats.2019.00315 (2019).

14 Yoshioka, T., Tsubota, T., Tashiro, K., Jouraku, A. & Kameda, T. A Study of the Extraordinarily Strong and Tough Silk Produced by Bagworms. *Nat Comm* **10**, 1469, doi: 10.1038/s41467-019-09359-3 (2019).

15 Riekel, C. & Vollrath, F. Spider Silk Fibre Extrusion: Combined Wide- and Small-Angle X-Ray Microdiffraction Experiments. *Int. J. Biol. Macrom.* **29**, 203-210, doi: 10.1016/s0141-8130(01)00166-0 (2001).

16 Mueller, M. *et al.* Direct Observation of Microfibril Arrangement in Single Native Cellulose Fiber by Microbeam Small-Angle X-Ray Scattering. *Macromolecules* **31**, 3953-3957, doi: 10.1021/ma980004c (1998).

17 Inouye, H., Fraser, P. E. & Kirschner, D. A. Structure of Beta-Crystallite Assemblies Formed by Alzheimer Beta-Amyloid Protein Analogues: Analysis by X-ray Diffraction. *Biophys J* **64**, 502-519, doi: 10.1016/S0006-3495(93)81393-6 (1993).

18 Marsh, R. E., Corey, R. B. & L.Pauling. The Structure of Tussah Silk Fibroin. *Acta Cryst.* **8**, 710-715, doi: 10.1107/S0365110X5500217X (1955).

19 Riekel, C., Burghammer, M. & Rosenthal, M. Skin-Core Morphology in Spider Flagelliform Silk. *APL* **115**, 123702, doi: 10.1063/1.5110268 (2019).

20 Foelix, R. F. *Biology of Spiders*. (Oxford University Press, 2011).

21 Hayashi, C. Y., Shipley, N. H. & Lewis, R. V. Hypothesis that Correlate the Sequence, Structure, and Mechanical Properties of Spider Silk Proteins. *Int. J. of Biol. Macrom.* **24**, 271-275, doi: 10.1016/s0141-8130(98)00089-0 (1999).

22 Blackledge, T. A., Kuntner, M. & Agnarsson, I. The Form and Function of Spider Orb Webs: Evolution from Silk to Ecosystems. *Adv. Insect Phys.* **41**, 175-262, doi: 10.1007/978-3-642-33989-9 (2011).

23 Guinea, G. V. *et al.* Minor Ampullate Silks from *Nephila* and *Argiope* Spiders: Tensile Properties and Microstructural Characterization. *Biomacromolecules* **13**, 2087-2098, doi: 10.1021/bm3004644 (2012).

24 Riekel, C., Madsen, B., Knight, D. & Vollrath, F. X-Ray Diffraction on Spider Silk during Controlled Extrusion under a Synchrotron Radiation X-Ray Beam. *Biomacromolecules* **1**, 622-626, doi: 10.1021/bm000047c (2000).

25 Sampath, S. *et al.* X-Ray Diffraction Study of Nanocrystalline and Amorphous Structure within Major and Minor Ampullate Dragline Spider Silks. *Soft Matter* **8**, 6713-6722, doi: 10.1039/C2SM25373A (2012).

26 Riekel, C., Craig, C. L., Burghammer, M. & Müller, M. Microstructural Homogeneity of Support Silk Spun by *Eriophora Fuliginea* (C.L. Koch) Determined by Scanning X-Ray Microdiffraction. *Naturwissenschaften* **88**, 67-72, doi: 10.1007/s001140000197 (2001).

27 Craig, C. L. *Spiderwebs and Silk: Tracing Evolution From Molecules to Genes to Phenotypes*. (Oxford University Press, 2003).

28 Crick, F. H. C. & Rich, A. Structure of Polyglycine II. *Nature* **176**, 780-781, doi: 10.1038/176780a0 (1955).
